# Subject Index: Volume 12

**DOI:** 10.1111/jdi.13731

**Published:** 2021-12-13

β‐Cell failure

β‐Cell failure in diabetes: Common susceptibility and mechanisms shared between type 1 and type 2 diabetes, Ikegami 1526–1539

β‐Cell mass

Glucokinase is required for high‐starch diet‐induced β‐cell mass expansion in mice, Tsuchida 1545–1554

β‐Cells

Islet β‐cells physiological difference study of old and young mice based on single‐cell transcriptomics, Zheng 1775–1783

Abnormal glucose tolerance

Nonalcoholic fatty liver disease is a risk factor for glucose intolerance onset in men regardless of alanine aminotransferase status, Miyake 1890–1898

Acetylation

SIRT7 regulates lipogenesis in adipocytes through deacetylation of PPARγ2, Akter 1765–1774

Acute myocardial infarction

Admission hyperglycemia as an independent predictor of long‐term prognosis in acute myocardial infarction patients without diabetes: A retrospective study, Cui 1244–1251
Islet microangiopathy and augmented β‐cell loss in Japanese non‐obese type 2 diabetes patients who died of acute myocardial infarction, Takahashi 2149–2161

Adenosine monophosphate‐activated protein kinases

Effect of adenosine monophosphate‐activated protein kinase–p53–Krüppel‐like factor 2a pathway in hyperglycemia‐induced cardiac remodeling in adult zebrafish, Wang 320–333

Adherence

Association between nutritional guidance or ophthalmological examination and discontinuation of physician visits in patients with newly diagnosed diabetes: A retrospective cohort study using a nationwide database, Akira Okada 1619–1631
Association between Grit Scales and adherence to regular hospital visits among Japanese patients with type 2 diabetes: Prospective observational study, Nakanishi 2259–2262

Adipocyte

Growth arrest‐specific 6 modulates adiponectin expression and insulin resistance in adipose tissue, Su 485–492
N4BP2L1 interacts with dynactin and contributes to GLUT4 trafficking and glucose uptake in adipocytes, Watanabe 1958–1966

Adipogenesis

Epigenetic regulation of N6‐methyladenosine modifications in obesity, Sun 1306–1315

Adiponectin

Inverse correlation between serum high‐molecular‐weight adiponectin and proinsulin level in a Japanese population: The Dynamics of Lifestyle and Neighborhood Community on Health Study, Nakamura 63–66
Growth arrest‐specific 6 modulates adiponectin expression and insulin resistance in adipose tissue, Su 485–492

Admission hyperglycemia

Admission hyperglycemia as an independent predictor of long‐term prognosis in acute myocardial infarction patients without diabetes: A retrospective study, Cui 1244–1251

Adverse outcome

Adverse obstetric outcomes in early‐diagnosed gestational diabetes mellitus: The Japan Environment and Children’s Study, Kyozuka 2071–2079

Age

Different interaction of onset age and duration of type 1 diabetes on the dynamics of autoantibodies to insulinoma‐associated antigen‐2 and zinc transporter 8, Kawasaki 510–515

Albuminuria

Plasma heparin cofactor II activity is inversely associated with albuminuria and its annual deterioration in patients with diabetes, Hara 2172–2182

Alcohol

Combination of alcohol and glucose consumption as a risk to induce reactive hypoglycemia, Oba‐Yamamoto 651–657

Alcohol drinking

Moderate alcohol consumption is associated with impaired insulin secretion and fasting glucose in non‐obese non‐diabetic men, Miyagi 869–876

Aldehyde dehydrogenase 2

Prospective study: Aldehyde dehydrogenase 2 gene is associated with cardio‐cerebrovascular complications in type 2 diabetes patients, He 1845–1854

Alginate

Microencapsulation of cells and molecular therapy of type 1 diabetes mellitus: The actual state and future perspectives between promise and progress, Basta 301–309

All‐cause mortality

Persons with type 2 diabetes and high insulin persistence were associated with a lower risk of mortality: A nationwide retrospective cohort study, Yen 146–154

Amino acids

Observational study of diabetes inpatients with gas chromatography/mass spectrometry‐based non‐target metabolomic analysis, Taya 2232–2241

Angiogenesis

Long non‐coding ribonucleic acid urothelial carcinoma‐associated 1 promotes high glucose‐induced human retinal endothelial cells angiogenesis through regulating micro‐ribonucleic acid‐624‐3p/vascular endothelial growth factor C, Yan 1948–1957

Anthropometric

Anthropometry did not approach adequate predictive accuracies for detecting elevated fasting plasma glucose or hemoglobin A1c levels among Chinese children aged 7–9 years, You 781–789

Annual estimated glomerular filtration rate decline

Sodium–glucose cotransporter 2 inhibitors in type 2 diabetes patients with renal function impairment slow the annual renal function decline, in a real clinical practice, Hirai 1577–1585

Antidiabetic drugs

Clinical inertia in patients with type 2 diabetes treated with oral antidiabetic drugs: Results from a Japanese cohort study (JDDM53), Maegawa 374–381

Antidiabetes agents

Effectiveness of antidiabetic agents for treatment of gestational diabetes: A methodological quality assessment of meta‐analyses and network meta‐analysis, Yarandi 2247–2258

Antiplatelet strategy

Antiplatelet strategy in primary and secondary prevention of cardiovascular disease in patients with type 2 diabetes mellitus: A perspective from the guideline appraisal, Liu 99–108

Arab women

Efficacy of lifestyle intervention program for Arab women with prediabetes using social media as an alternative platform of delivery, Al‐Hamdan 1872–1880

Arterial stiffness

Lower intake of saturated fatty acids is associated with persistently higher arterial stiffness in patients with type 2 diabetes, Mita 226–233

Artificial intelligence

Imaging the eye and its relevance to diabetes care, Quinn 897–908

Asians

East Asian diet‐mimicking diet plan based on the Mediterranean diet and the Dietary Approaches to Stop Hypertension diet in adults with type 2 diabetes: A randomized controlled trial, Jin 357–364
Sex differences in the association between fatty liver and type 2 diabetes incidence in non‐obese Japanese: A retrospective cohort study, Narisada 1480–1489

Asian subjects

Glucose‐lowering action through targeting islet dysfunction in type 2 diabetes: Focus on dipeptidyl peptidase‐4 inhibition, Ahrén 1128–1135

Autoantibodies

Different interaction of onset age and duration of type 1 diabetes on the dynamics of autoantibodies to insulinoma‐associated antigen‐2 and zinc transporter 8, Kawasaki 510–515
Diagnostic value of combined islet antigen‐reactive T cells and autoantibodies assays for type 1 diabetes mellitus, Tang 963–969

Autologous fibroblast

Combination therapy of trichloroacetic acid, human autologous fibroblast injection and fibroblast seeded microfibrous collagen scaffold as a novel treatment for osteomyelitis diabetic foot ulcer, Nilforoushzadeh 1112–1117

Autonomic nervous system diseases

Insulin resistance is independently associated with cardiovascular autonomic neuropathy in type 2 diabetes, Liu 1651–1662

Bacterial meningitis

Pyogenic psoas abscess on the dorsal side, and bacterial meningitis and spinal epidural abscess on the ventral side, both of which were induced by spontaneous discitis in a patient with diabetes mellitus: A case report, Horiya 1301–1305

Bangladesh

Health‐related quality of life and its predictors among the type 2 diabetes population of Bangladesh: A nation‐wide cross‐sectional study, Barua 277–285

Biliary diversion

Biliary diversion increases resting energy expenditure leading to decreased blood glucose level in mice with type 2 diabetes, Yin 931–939

Bilirubin

Bilirubin is inversely related to diabetic peripheral neuropathy assessed by sural nerve conduction study, Abe 2028–2035

Bioimpedance phase angle

Mediation effect of the duration of diabetes mellitus on the decrease in bioimpedance phase angles in ethnically Korean people: A multicenter clinical study, Jun 790–802

Biomarker

Fractional excretion of tumor necrosis factor receptor 1 and 2 in patients with type 2 diabetes and normal renal function, Gohda 382–389

Birth cohort study

Adverse obstetric outcomes in early‐diagnosed gestational diabetes mellitus: The Japan Environment and Children’s Study, Kyozuka 2071–2079

Birthweight

Association between neonatal birthweight and risk of maternal glucose intolerance after gestational diabetes mellitus, Li 425–433

Blood glucose

Transition of blood glucose level in a patient with pregnancy‐associated fulminant type 1 diabetes mellitus, Ichikawa 894–896

Blood lipids and lipid ratios

Sex‐specific differences in blood lipids and lipid ratios in type 2 diabetic foot patients, Yin, 2203–2211

Blood pressure

Blood pressure after treatment with sodium–glucose cotransporter 2 inhibitors influences renal composite outcome: Analysis using propensity score‐matched models, Kobayashi, 74–81
Sodium–glucose cotransporter 2 inhibitor‐induced reduction in the mean arterial pressure improved renal composite outcomes in type 2 diabetes mellitus patients with chronic kidney disease: A propensity score‐matched model analysis in Japan, Kobayashi 1408–1416

Body composition

Effects of sedentary behavior and daily walking steps on body mass index and body composition: Prospective observational study using outpatient clinical data of Japanese patients with type 2 diabetes, Nakanishi 1732–1738

Body fluid volume

Impact of extracellular‐to‐intracellular fluid volume ratio on albuminuria in patients with type 2 diabetes: A cross‐sectional and longitudinal cohort study, Nakajima 1202–1211

Body mass index

Significance of body mass index for diagnosing sarcopenia is equivalent to slow gait speed in Japanese individuals with type 2 diabetes: Cross‐sectional study using outpatient clinical data, Nakanishi 417–424

Body size

Moderate alcohol consumption is associated with impaired insulin secretion and fasting glucose in non‐obese non‐diabetic men, Miyagi 869–876

Bone metabolism

Effects of ipragliflozin versus metformin in combination with sitagliptin on bone and muscle in Japanese patients with type 2 diabetes mellitus: Subanalysis of a prospective, randomized, controlled study (PRIME‐V study), Koshizaka 200–206

Bone mineral density

Lower bone mineral density and higher bone resorption marker levels in premenopausal women with type 1 diabetes in Japan, Yoshioka 1689–1696

Bromocriptine

Rapid and dramatic glucose‐lowering effect of bromocriptine in an inadequately controlled type 2 diabetes patient with prolactinoma, Igata 668–671

Brown adipose tissue

Kruppel‐like factor 15 regulates fuel switching between glucose and fatty acids in brown adipocytes, Nabatame 1144–1151

C1q TNF‐related protein 9

Association of serum CTRP9 levels with cardiac autonomic neuropathy in patients with type 2 diabetes mellitus, Yang 1442–1451

C22:6

Potential biomarker in serum for predicting susceptibility to type 2 diabetes mellitus: Free fatty acid 22:6, Ma 950–962

Ca2+ transients

Calcium signaling in endocardial and epicardial ventricular myocytes from streptozotocin‐induced diabetic rats, Al Kury 493–500

Canagliflozin

Simplification of complex insulin regimens using canagliflozin or liraglutide in patients with well‐controlled type 2 diabetes: A 24‐week randomized controlled trial, Ando 1816–1826

Capillaries

Association of crossing capillaries in the finger nailfold with diabetic retinopathy in type 2 diabetes mellitus, Shikama 1007–1014

Carboxymethyl arginine

Glyoxal‐induced formation of advanced glycation end‐products in type 1 collagen decreases both its strength and flexibility *in vitro*, Kitamura 1555–1559

Cardiac autonomic neuropathy

Association of serum CTRP9 levels with cardiac autonomic neuropathy in patients with type 2 diabetes mellitus, Yang 1442–1451

Cardio‐cerebrovascular complications

Prospective study: Aldehyde dehydrogenase 2 gene is associated with cardio‐cerebrovascular complications in type 2 diabetes patients, He 1845–1854

Cardiorespiratory fitness

Predictors of the maximal oxygen consumption in adult patients with type 1 diabetes treated with personal insulin pumps, Matejko 1377–1385

Cardiometabolic‐renal risk factors

Association of hip fractures with cardiometabolic‐renal risk factors in Southern Chinese patients with type 2 diabetes – the Hong Kong Diabetes Register, Cheung 1739–1748

Cardiovascular autonomic neuropathy

Feasibility of combining heart rate variability and electrochemical skin conductance as screening and severity evaluation of cardiovascular autonomic neuropathy in type 2 diabetes, Lai 1671–1679

Cardiovascular disease

Antiplatelet strategy in primary and secondary prevention of cardiovascular disease in patients with type 2 diabetes mellitus: A perspective from the guideline appraisal, Liu 99–108
Persons with type 2 diabetes and high insulin persistence were associated with a lower risk of mortality: A nationwide retrospective cohort study, Yen 146–154
Incidence of adverse cardiovascular events in type 2 diabetes mellitus patients after initiation of glucose‐lowering agents: A population‐based community study from the Shizuoka Kokuho database, Kohsaka 1452–1461

Cardiovascular disease

Association of visit‐to‐visit glycemic variability with risk of cardiovascular diseases in high‐risk Japanese patients with type 2 diabetes: A subanalysis of the EMPATHY trial, Sato 2190–2196

Cardiovascular event

Sodium–glucose cotransporter 2 inhibitors compared with other glucose‐lowering drugs in Japan: Subanalyses of the CVD‐REAL 2 Study, Kohsaka 67–73
Impact of daily glucose fluctuations on cardiovascular outcomes after percutaneous coronary intervention for patients with stable coronary artery disease undergoing lipid‐lowering therapy, Yamamoto 1015–1024

Cardiovascular events

Association between glycemic control and cardiovascular events in older Japanese adults with diabetes mellitus: An analysis of the Japanese medical administrative database, Yokote 2036–2045

Cardiovascular risk factors

Metabolic syndrome coexists with adult Léri–Weill dyschondrosteosis: A case report, Wang 446–449

Carpal tunnel syndrome

Diabetic polyneuropathy and carpal tunnel syndrome together affect hand strength, tactile sensation and dexterity in diabetes patients, Zhang 2010–2018

Case–control study

Dipeptidyl peptidase‐4 inhibitor might exacerbate Graves’ disease: A multicenter observational case–control study, Sekizaki 1978–1982

Cell shrinkage

Association of mean corpuscular volume with sarcopenia and visceral obesity in individuals without anemia, Tanaka 1287–1292

Charcot–Marie–Tooth disease

Diabetes coexistent with Charcot–Marie–Tooth disease presenting as a recurrent foot ulcer misdiagnosed as diabetic foot: A case report, Yan 2099–2101

Children

Anthropometry did not approach adequate predictive accuracies for detecting elevated fasting plasma glucose or hemoglobin A1c levels among Chinese children aged 7–9 years, You 781–789

China

Phase III, randomized, double‐blind, placebo‐controlled study to evaluate the efficacy and safety of teneligliptin monotherapy in Chinese patients with type 2 diabetes mellitus inadequately controlled with diet and exercise, Ji 537–545

Chinese

Recognition of maturity‐onset diabetes of the young in China, Liang 501–509

Chinese adults

Low‐density lipoprotein cholesterol : high‐density lipoprotein cholesterol ratio is associated with incident diabetes in Chinese adults: A retrospective cohort study, Wei 91–98

Chronic kidney disease

Changing epidemiology of chronic kidney disease as a result of type 2 diabetes mellitus from 1990 to 2017: Estimates from Global Burden of Disease 2017, Li 346–356
Sodium–glucose cotransporter 2 inhibitor‐induced reduction in the mean arterial pressure improved renal composite outcomes in type 2 diabetes mellitus patients with chronic kidney disease: A propensity score‐matched model analysis in Japan, Kobayashi 1408–1416

CIDEC

CIDEC silencing attenuates diabetic nephropathy via inhibiting apoptosis and promoting autophagy, Zheng 1336–1345

Classification of diabetes

Identification of two novel subgroups in patients with diabetes mellitus and their association with clinical outcomes: A two‐step cluster analysis, Xiong 1346–1358

Clinical epidemiology

Association between nutritional guidance or ophthalmological examination and discontinuation of physician visits in patients with newly diagnosed diabetes: A retrospective cohort study using a nationwide database, Akira Okada 1619–1631

Cluster analysis

Identification of two novel subgroups in patients with diabetes mellitus and their association with clinical outcomes: A two‐step cluster analysis, Xiong 1346–1358

Coagulation disorders

Clinical profiles of hyperglycemic crises: A single‐center retrospective study from Japan, Nishikawa 1359–1366

Coefficient of variation

Defining the target value of the coefficient of variation by continuous glucose monitoring in Chinese people with diabetes, Mo 1025–1034

Cohort study

Dietary fiber intake and risk of type 2 diabetes in a general Japanese population: The Hisayama Study, Kimura 527–536
Development of a clinical risk score for incident diabetes: A 10‐year prospective cohort study, Oh 610–618
Frequency of consumption of balanced meals, bodyweight gain and incident risk of glucose intolerance in Japanese men and women: A cohort study, Sakurai 763–770
Association between serum prostate‐specific antigen concentrations and the risk of developing type 2 diabetes mellitus in Chinese men: A cohort study, Huang 1560–1568
Combination of disease duration‐to‐age at diagnosis and hemoglobin A1c‐to‐serum C‐peptide reactivity ratios predicts patient response to glucose‐lowering medication in type 2 diabetes: A retrospective cohort study across Japan (JDDM59), Kanatsuka 1967–1977

Collective risk factor

Combination of disease duration‐to‐age at diagnosis and hemoglobin A1c‐to‐serum C‐peptide reactivity ratios predicts patient response to glucose‐lowering medication in type 2 diabetes: A retrospective cohort study across Japan (JDDM59), Kanatsuka 1967–1977

Commentary

Glucagon regulates lipolysis and fatty acid oxidation through inositol triphosphate receptor 1 in the liver, Hayashi 32–34
Invincible β‐cells in type 1 diabetes, Choe 137–139
Diagnostic strategy for diabetic polyneuropathy: Focus on nerve fiber type and magnetic resonance neurography, Sasaki 140–142
Sodium–glucose cotransporter 2 inhibitors: A drug with antidiabetic and cardioprotective properties, Li 310–312
Commentary on risk factors for first and subsequent cardiovascular disease events in type 1 diabetes: The Diabetes Control and Complications Trial/Epidemiology of Diabetes Interventions and Complications study, Lin 313–316
Avoid clinical inertia: Importance of asking and advising patients with diabetes who smoke about quitting, Yatsuya 317–319
Microbe‐associated metabolites as targets for incident type 2 diabetes, Hashimoto 476–478
Is metformin a miracle or a menace in COVID‐19 patients with type 2 diabetes?, Lui 479–481
Classical molecule in diabetic kidney hypertrophy is linked to defects in self‐eating through fine‐tuning, Takagaki 686–688
Body‐weight‐independent glucose‐lowering effect of the β3‐adrenergic receptor agonist mirabegron in humans, Waki 689–690
Patch‐seq shows the heterogeneity of pancreatic islet cells, Asahara 691–693

Continuity of patient care

Synergy between the pay‐for‐performance scheme and better physician–patient relationship might reduce the risk of retinopathy in patients with type 2 diabetes, Chiou 819–827
T helper 17 cells: A new actor on the stage of type 2 diabetes and aging?, Chang 909–913
Effects of glucagon‐like peptide‐1 receptor agonists on kidney function and safety in type 2 diabetes patients, Kim 914–916
Cell‐autonomous defects contribute to insulin resistance in skeletal muscle, Fujita 1136–1137
Dual‐specificity phosphatase 8: A gatekeeper in hypothalamic control of glucose metabolism in males, Kaneko 1138–1140
Intensive risk factor management and cardiovascular autonomic neuropathy in type 2 diabetes in the Action to Control Cardiovascular Risk in Diabetes trial: A post‐hoc analysis, Aso 1316‐1318
The gut microbial metabolite imidazole propionate inhibits metformin action, Sugawara 1319–1321
Inceptor intercepts insulin signaling in pancreatic β‐cells, Watada 1540–1541
Fighting the uphill battle to cure type 1 diabetes, Lee 1542–1544
Eating whole fruit, not drinking fruit juice, may reduce the risk of type 2 diabetes mellitus, Seino 1759–1761
Can we catch the second loach employing BACE1 inhibition, even as the first one might be escaping?, Ono 1942–1943
Commentary on Intraglumerular dysfunction predicts kidney failure in the type 2 diabetes Jun Wada 2124 Pancreatic β‐cell fate in subjects with COVID‐19, Shirakawa 2126–2125
Pancreatic β‐cell fate in subjects with COVID‐19, Shirakawa 2126–2128

Continuous glucose monitoring

Relationships between time in range, glycemic variability including hypoglycemia and types of diabetes therapy in Japanese patients with type 2 diabetes mellitus: Hyogo Diabetes Hypoglycemia Cognition Complications study, Kuroda 244–253
Is it possible to predict the onset of nocturnal asymptomatic hypoglycemia in patients with type 1 diabetes receiving insulin degludec? Potential role of previous day and next morning glucose values, Takahashi 365–373
Rapid and dramatic glucose‐lowering effect of bromocriptine in an inadequately controlled type 2 diabetes patient with prolactinoma, Igata 668–671
Association of time in range, as assessed by continuous glucose monitoring, with painful diabetic polyneuropathy, Yang 828–836
Association of time in range with hemoglobin A1c, glycated albumin and 1,5‐anhydro‐d‐glucitol, Ohigashi 940–949
Defining the target value of the coefficient of variation by continuous glucose monitoring in Chinese people with diabetes, Mo 1025–1034
Glycemic control in children and teenagers with type 1 diabetes around lockdown for COVID‐19: A continuous glucose monitoring‐based observational study, Wu 1708–1717

Corneal confocal microscopy

Diagnostic utility of corneal confocal microscopy in type 2 diabetic peripheral neuropathy, Wang 574–582
Painful diabetic neuropathy is associated with increased nerve regeneration in patients with type 2 diabetes undergoing intensive glycemic control, Ponirakis 1642–1650
Insulin resistance limits corneal nerve regeneration in patients with type 2 diabetes undergoing intensive glycemic control, Ponirakis 2002–2009

Corneal confocal microscopy

Corneal confocal microscopy: A useful tool for diagnosis of small fiber neuropathy in type 2 diabetes, Jin 2183–2189

Coronavirus disease 2019

Glycemic control before admission is an important determinant of prognosis in patients with coronavirus disease 2019, Liu 1064–1073
Clinical utility of 1‐month postpartum random plasma glucose and glycated hemoglobin combined with pre‐pregnancy body mass index for detecting postpartum glucose intolerance in Japanese women with gestational diabetes, Sugiyama 2242–2246

Coronary disease

Reduced leukocyte telomere lengths and sirtuin 1 gene expression in long‐term survivors of type 1 diabetes: A Dialong substudy, Opstad 1183–1192

COVID‐19

Young people with type 1 diabetes on insulin pump therapy could fast safely during COVID–19 pandemic Ramadan: A telemonitoring experience in Bangladesh, Zabeen 1060–1063
Glycemic control in children and teenagers with type 1 diabetes around lockdown for COVID‐19: A continuous glucose monitoring‐based observational study, Wu 1708–1717
Lifestyle changes as a result of COVID‐19 containment measures: Bodyweight and glycemic control in patients with diabetes in the Japanese declaration of a state of emergency, Tanaka 1718–1722

C‐peptide

Impact of endogenous insulin secretion on the improvement of glucose variability in Japanese patients with type 2 diabetes treated with canagliflozin plus teneligliptin, Miya 1395–1399

C‐X‐C motif chemokine ligand 14

Serum C‐X‐C motif chemokine ligand 14 levels are associated with serum C‐peptide and fatty liver index in type 2 diabetes mellitus patients, Matsushita 1042–1049

*dachshund b*

Investigating the role of *dachshund b* in the development of the pancreatic islet in zebrafish, Yang 710–727

Dairy products

Lower intake of saturated fatty acids is associated with persistently higher arterial stiffness in patients with type 2 diabetes, Mita 226–233

Denosumab

Beneficial effects of switching to denosumab from bisphosphonates or selective estrogen receptor modulators in postmenopausal women with type 2 diabetes and osteopenia/osteoporosis, Miyoshi 1293–1300

Diabetes

Loss of metabolic flexibility as a result of overexpression of pyruvate dehydrogenase kinases in muscle, liver and the immune system: Therapeutic targets in metabolic diseases, Jeon 21–31
Prediabetes and long‐term outcomes in patients with three‐vessel coronary artery disease: A large single‐center cohort study, Yuan 409–416
Serum vaspin levels are positively associated with diabetic retinopathy in patients with type 2 diabetes mellitus, Yang 566–573
Increased body fat mass reduces the association between fructosamine and glycated hemoglobin in obese type 2 diabetes patients, Vergès 619–624
Irisin plays an important role in the outcomes of newly diagnosed prediabetes in adults in Guiyang, China, He 747–755
Asymptomatic postprandial hypotension in patients with diabetes: The KAMOGAWA‐HBP study, Kitae 837–844
Effects of mindfulness‐based intervention on glycemic control and psychological outcomes in people with diabetes: A systematic review and meta‐analysis, Ni 1092–1103
Triglyceride to high‐density lipoprotein cholesterol ratio variability and incident diabetes: A 7‐year prospective study in a Chinese population, Cai 1864–1871
Midlife and late‐life diabetes and sarcopenia in a general older Japanese population: The Hisayama Study, Nakamura 1899–1907
Genetic susceptibility, family history of diabetes and healthy lifestyle factors in relation to diabetes: A gene–environment interaction analysis in Chinese adults, Ye 2089–2098
Effect of glycemic gap upon mortality in critically ill patients with diabetes, Lou 2212–2220

Diabetes Card System Program

Effects of physician’s diabetes self‐management education using Japan Association of Diabetes Education and Care Diabetes Education Card System Program and a self‐monitoring of blood glucose readings analyzer in individuals with type 2 diabetes: An exploratory, open‐labeled, prospective randomized clinical trial, Tanaka 2221–2231

Diabetes complications

Synergy between the pay‐for‐performance scheme and better physician–patient relationship might reduce the risk of retinopathy in patients with type 2 diabetes, Chiou 819–827

Diabetes mellitus

Clinical and genetic analysis in a Chinese cohort of children and adolescents with diabetes/persistent hyperglycemia, Ding 48–62
East Asian diet‐mimicking diet plan based on the Mediterranean diet and the Dietary Approaches to Stop Hypertension diet in adults with type 2 diabetes: A randomized controlled trial, Jin 357–364
Maternal and paternal histories differentially influence risks for diabetes, insulin secretion and insulin resistance in a Chinese population, Kong 434–445
Associations between sarcopenia and white matter alterations in older adults with diabetes mellitus: A diffusion tensor imaging study, Tamura 633–640
Clinical and genetic determinants of urinary glucose excretion in patients with diabetes mellitus, Monobe 728–737
Trends in the effects of pre‐transplant diabetes on mortality and cardiovascular events after kidney transplantation, Jeon 811–818
Risk factors for abnormal postpartum glycemic states in women diagnosed with gestational diabetes by the International Association of Diabetes and Pregnancy Study Groups criteria, Hung 859–868
High urinary glucose is associated with improved renal prognosis in patients with diabetes mellitus, Itano 998–1006
Epigenetic changes caused by diabetes and their potential role in the development of periodontitis, Li 1326–1335
Metformin as a potential protective therapy against tuberculosis in patients with diabetes mellitus: A retrospective cohort study in a single teaching hospital, Fu 1603–1609
Elevated serum pentosidine is independently associated with the high prevalence of sarcopenia in Chinese middle‐aged and elderly men with type 2 diabetes mellitus, Zhang 2054–2061

Diabetes mellitus type 1

Sodium–glucose cotransporter inhibitors as add‐on therapy in addition to insulin for type 1 diabetes mellitus: A meta‐analysis of randomized controlled trials, Zou 546–556
Reduced leukocyte telomere lengths and sirtuin 1 gene expression in long‐term survivors of type 1 diabetes: A Dialong substudy, Opstad 1183–1192
Association between influenza and the incidence rate of new‐onset type 1 diabetes in Japan, Nishioka 1797–1804

Diabetes mellitus type 2

Clinical inertia in patients with type 2 diabetes treated with oral antidiabetic drugs: Results from a Japanese cohort study (JDDM53), Maegawa 374–381

Diabetes neuropathy

Prevalence and risk factors for diabetic neuropathy and painful diabetic neuropathy in primary and secondary healthcare in Qatar, Ponirakis 592–600

Diabetes prevention

Efficacy of lifestyle intervention program for Arab women with prediabetes using social media as an alternative platform of delivery, Al‐Hamdan 1872–1880

Diabetes self‐management education

Effects of physician’s diabetes self‐management education using Japan Association of Diabetes Education and Care Diabetes Education Card System Program and a self‐monitoring of blood glucose readings analyzer in individuals with type 2 diabetes: An exploratory, open‐labeled, prospective randomized clinical trial, Tanaka 2221–2231

Diabetes treatment

Observational study of diabetes inpatients with gas chromatography/mass spectrometry‐based non‐target metabolomic analysis, Taya 2232–2241

Diabetic cardiomyopathies

Effect of adenosine monophosphate‐activated protein kinase–p53–Krüppel‐like factor 2a pathway in hyperglycemia‐induced cardiac remodeling in adult zebrafish, Wang 320–333

Diabetic cardiomyopathy

High prevalence of fragmented QRS on electrocardiography in Japanese patients with diabetes irrespective of metabolic syndrome, Yagi 1680–1688

Diabetic complication

Role of O‐linked N‐acetylglucosamine in the homeostasis of metabolic organs, and its potential links with diabetes and its complications, Morino 130–136
Impaired cardiac and neurological function with mild hypophosphatemia during insulin therapy for diabetic ketoacidosis and marked improvement with phosphate supplementation: A case report, Yoshida 454–458

Diabetic foot

Diabetes coexistent with Charcot–Marie–Tooth disease presenting as a recurrent foot ulcer misdiagnosed as diabetic foot: A case report, Yan 2099–2101
Sex‐specific differences in blood lipids and lipid ratios in type 2 diabetic foot patients, Yin, 2203–2211

Diabetic foot ulcer

Combination therapy of trichloroacetic acid, human autologous fibroblast injection and fibroblast seeded microfibrous collagen scaffold as a novel treatment for osteomyelitis diabetic foot ulcer, Nilforoushzadeh 1112–1117

Diabetic ketoacidosis

Fractional excretion of tumor necrosis factor receptor 1 and 2 in patients with type 2 diabetes and normal renal function, Gohda 382–389
Clinical profiles of hyperglycemic crises: A single‐center retrospective study from Japan, Nishikawa 1359–1366
On‐label use of sodium–glucose cotransporter 2 inhibitors might increase the risk of diabetic ketoacidosis in patients with type 1 diabetes, Horii 1586–1593

Diabetic kidney disease

Randomized trial of an intensified, multifactorial intervention in patients with advanced‐stage diabetic kidney disease: Diabetic Nephropathy Remission and Regression Team Trial in Japan (DNETT‐Japan), Shikata 207–216
Impact of extracellular‐to‐intracellular fluid volume ratio on albuminuria in patients with type 2 diabetes: A cross‐sectional and longitudinal cohort study, Nakajima 1202–1211
One‐year estimated glomerular filtration rate decline as a risk factor of cardiovascular and renal end‐points in high‐risk Japanese patients, Meguro 1212–1219
Sodium–glucose cotransporter 2 inhibitors in type 2 diabetes patients with renal function impairment slow the annual renal function decline, in a real clinical practice, Hirai 1577–1585
Epidemiological characteristics of diabetic kidney disease in Taiwan, Wang 2112–2123

Diabetic myopathy

Nicorandil decreases oxidative stress in slow‐ and fast‐twitch muscle fibers of diabetic rats by improving the glutathione system functioning, Sánchez‐Duarte 1152–1161

Diabetic nephropathy

Blood pressure after treatment with sodium–glucose cotransporter 2 inhibitors influences renal composite outcome: Analysis using propensity score‐matched models, Kobayashi 74–81
Randomized trial of an intensified, multifactorial intervention in patients with advanced‐stage diabetic kidney disease: Diabetic Nephropathy Remission and Regression Team Trial in Japan (DNETT‐Japan), Shikata 207–216
Association between the triglyceride–glucose index and diabetic nephropathy in patients with type 2 diabetes: A cross‐sectional study, Liu 557–565
The PKM2 activator TEPP‐46 suppresses kidney fibrosis via inhibition of the EMT program and aberrant glycolysis associated with suppression of HIF‐1α accumulation, Liu 697–709
CIDEC silencing attenuates diabetic nephropathy via inhibiting apoptosis and promoting autophagy, Zheng 1336–1345
All‐cause and cardiovascular disease mortality in underweight patients with diabetic nephropathy: BioBank Japan cohort, Yokomichi 1425–1429

Diabetic neuropathies

Point‐of‐care nerve conduction device predicts the severity of diabetic polyneuropathy: A quantitative, but easy‐to‐use, prediction model, Kamiya 583–591
Neuroretinal dysfunction revealed by a flicker electroretinogram correlated with peripheral nerve dysfunction and parameters of atherosclerosis in patients with diabetes, Kawai 1236–1243
Bilirubin is inversely related to diabetic peripheral neuropathy assessed by sural nerve conduction study, Abe 2028–2035

Diabetic neuropathy

Loss of lower extremity muscle strength based on diabetic polyneuropathy in older patients with type 2 diabetes: Multicenter Survey of the Isometric Lower Extremity Strength in Type 2 Diabetes: Phase 2 study, Nomura 390–397

Diabetic peripheral neuropathy

Diagnostic utility of corneal confocal microscopy in type 2 diabetic peripheral neuropathy, Wang 574–582
Association of platelet count and plateletcrit with nerve conduction function and peripheral neuropathy in patients with type 2 diabetes mellitus, Qian 1835–1844
Insulin resistance limits corneal nerve regeneration in patients with type 2 diabetes undergoing intensive glycemic control, Ponirakis 2002–2009
Liver fibrosis is independently associated with diabetic peripheral neuropathy in type 2 diabetes mellitus, Huang 2019–2027

Diabetic polyneuropathies

Alterations of retinal thickness measured by optical coherence tomography correlate with neurophysiological measures in diabetic polyneuropathy, Yamada 1430–1441

Diabetic polyneuropathy

Bilateral atrophy of the extensor digitorum brevis muscle might be a useful sign for diagnosing diabetic polyneuropathy in Japanese men who do not sit in the traditional “*seiza*” style, Kishimoto 398–408
Current concepts in the management of diabetic polyneuropathy, Ziegler 464–475
Quantitative vibration perception threshold in assessing diabetic polyneuropathy: Should the cut‐off value be adjusted for Chinese individuals with type 2 diabetes?, Liu 1663–1670
Falls and fractures associated with type 2 diabetic polyneuropathy: A cross‐sectional nationwide questionnaire study, Khan 1827–1834
Diabetic polyneuropathy and carpal tunnel syndrome together affect hand strength, tactile sensation and dexterity in diabetes patients, Zhang 2010–2018

Diabetic retinopathy

Serum vaspin levels are positively associated with diabetic retinopathy in patients with type 2 diabetes mellitus, Yang 566–573
Imaging the eye and its relevance to diabetes care, Quinn 897–908
Association of crossing capillaries in the finger nailfold with diabetic retinopathy in type 2 diabetes mellitus, Shikama 1007–1014
Normal parathyroid hormone and non‐proliferative diabetic retinopathy in patients with type 2 diabetes, Sun 1220–1227
Association of non‐alcoholic fatty liver disease with diabetic retinopathy in type 2 diabetic patients: A meta‐analysis of observational studies, Song 1471–1479
Long non‐coding ribonucleic acid urothelial carcinoma‐associated 1 promotes high glucose‐induced human retinal endothelial cells angiogenesis through regulating micro‐ribonucleic acid‐624‐3p/vascular endothelial growth factor C, Yan 1948–1957

Diabetic retinopathy screening

Screening for diabetic retinopathy with different levels of financial incentive in a randomized controlled trial, Lian 1632–1641

Diabetic vascular complications

Associations between urinary 6‐sulfatoxymelatonin excretion and diabetic vascular complications or arteriosclerosis in patients with type 2 diabetes, Tanaka 601–609

Diagnosis

Current concepts in the management of diabetic polyneuropathy, Ziegler 464–475
Prevalence and risk factors for diabetic neuropathy and painful diabetic neuropathy in primary and secondary healthcare in Qatar, Ponirakis 592–600

Diagnostic model

Diagnostic value of dysregulated microribonucleic acids in the placenta and circulating exosomes in gestational diabetes mellitus, Zhang 1490–1500

Dichorionic twins

Risk factors and adverse maternal and perinatal outcomes for women with dichorionic twin pregnancies complicated by gestational diabetes mellitus: A retrospective cross‐sectional study, Hung 1083–1091

Dietary fiber

Dietary fiber intake and risk of type 2 diabetes in a general Japanese population: The Hisayama Study, Kimura 527–536

Dietary intervention

Potassium supplementation blunts the effects of high salt intake on serum retinol‐binding protein 4 levels in healthy individuals, Liu 658–663

Diffusion tensor imaging

Associations between sarcopenia and white matter alterations in older adults with diabetes mellitus: A diffusion tensor imaging study, Tamura 633–640
Dipeptidyl peptidase‐4 inhibitor, anagliptin, alters hepatic insulin clearance in relation to the glycemic status in Japanese individuals with type 2 diabetes, Abe, 1805–1815

Dipeptidyl peptidase‐4

Dipeptidyl peptidase‐4 inhibitor might exacerbate Graves’ disease: A multicenter observational case–control study, Sekizaki 1978–1982

Dipeptidyl peptidase‐4 inhibition

Glucose‐lowering action through targeting islet dysfunction in type 2 diabetes: Focus on dipeptidyl peptidase‐4 inhibition, Ahrén 1128–1135

Dipeptidyl peptidase‐4 inhibitor

Dipeptidyl peptidase‐4 inhibitor, anagliptin, alters hepatic insulin clearance in relation to the glycemic status in Japanese individuals with type 2 diabetes, Abe, 1805–1815

Dipeptidyl peptidase‐4 inhibitors

Phase III, randomized, double‐blind, placebo‐controlled study to evaluate the efficacy and safety of teneligliptin monotherapy in Chinese patients with type 2 diabetes mellitus inadequately controlled with diet and exercise, Ji 537–545

Duration of diabetes mellitus

Mediation effect of the duration of diabetes mellitus on the decrease in bioimpedance phase angles in ethnically Korean people: A multicenter clinical study, Jun 790–802

Dynapenia

High prevalence and clinical impact of dynapenia and sarcopenia in Japanese patients with type 1 and type 2 diabetes: Findings from the Impact of Diabetes Mellitus on Dynapenia study, Mori 1050–1059

Eating behavior

Lifestyle changes as a result of COVID‐19 containment measures: Bodyweight and glycemic control in patients with diabetes in the Japanese declaration of a state of emergency, Tanaka 1718–1722

Edema

Case of type 2 diabetes mellitus with edema resulting in subcutaneous insulin resistance syndrome Ying Hu, Qiongwen Zhu, Xiuli Yang, Jieping Yan, and Jiana Shi 2267–2270

Editorial

Carbohydrate‐induced weight gain models for diabetes research: Contribution of incretins and parasympathetic signal Seino, 3–5
Being caught in the perfect storm of a diabetes epidemic and the COVID‐19 pandemic: What should we do for our patients?, Cho 297–300
The COVID‐19 world – Are we there yet?, Hamamoto 1125‐1127
Challenges of optimizing insulin therapy for patients with type 2 diabetes mellitus, Yen 1523–1525
New mediators in diabetes pathogenesis: Exosomes and metabolites, Lee 1931–1933
Autonomic dysfunction, diabetes and metabolic syndrome, Yu 2108–2111

Effects

Sodium–glucose cotransporter inhibitors as add‐on therapy in addition to insulin for type 1 diabetes mellitus: A meta‐analysis of randomized controlled trials, Zou 546–556

Electrocardiography

High prevalence of fragmented QRS on electrocardiography in Japanese patients with diabetes irrespective of metabolic syndrome, Yagi 1680–1688

Electrochemical skin conductance

Feasibility of combining heart rate variability and electrochemical skin conductance as screening and severity evaluation of cardiovascular autonomic neuropathy in type 2 diabetes, Lai 1671–1679

Electromyography

Point‐of‐care nerve conduction device predicts the severity of diabetic polyneuropathy: A quantitative, but easy‐to‐use, prediction model, Kamiya 583–591

Electroretinography

Neuroretinal dysfunction revealed by a flicker electroretinogram correlated with peripheral nerve dysfunction and parameters of atherosclerosis in patients with diabetes, Kawai 1236–1243

Empagliflozin

Early detection of euglycemic ketoacidosis during thoracic surgery associated with empagliflozin in a patient with type 2 diabetes: A case report, Kitahara 664–667

EMT

The PKM2 activator TEPP‐46 suppresses kidney fibrosis via inhibition of the EMT program and aberrant glycolysis associated with suppression of HIF‐1α accumulation, Liu 697–709

Endoplasmic reticulum stress

β‐Cell failure in diabetes: Common susceptibility and mechanisms shared between type 1 and type 2 diabetes, Ikegami 1526–1539

Energy

Biliary diversion increases resting energy expenditure leading to decreased blood glucose level in mice with type 2 diabetes, Yin 931–939

Energy expenditure

Diet intake control is indispensable for the gluconeogenic response to sodium–glucose cotransporter 2 inhibition in male mice, Hashiuchi 35–47

Enzyme‐linked immunospot assay

Diagnostic value of combined islet antigen‐reactive T cells and autoantibodies assays for type 1 diabetes mellitus, Tang 963–969

Epidemiology

Epidemiology, genetic landscape and classifi cation of childhood diabetes mellitus in the State of Qatarm Haris 2141–2148

Epigenetics

Placental maternally expressed gene 3 differentially methylated region methylation profile is associated with maternal glucose concentration and newborn birthweight, Chen 1074–1082
Epigenetic changes caused by diabetes and their potential role in the development of periodontitis, Li 1326–1335

Estimated glomerular filtration rate

One‐year estimated glomerular filtration rate decline as a risk factor of cardiovascular and renal end‐points in high‐risk Japanese patients, Meguro 1212–1219

Ethnic groups

Cardiovascular events and mortality in people with and without type 2 diabetes: An observational study in a contemporary multi‐ethnic population, Coles 1175–1182

Exenatide

Painful diabetic neuropathy is associated with increased nerve regeneration in patients with type 2 diabetes undergoing intensive glycemic control, Ponirakis 1642–1650

Exosome

Diagnostic value of dysregulated microribonucleic acids in the placenta and circulating exosomes in gestational diabetes mellitus, Zhang 1490–1500

Expression quantitative trait locus

Novel association between a transient receptor potential cation channel subfamily M member 5 expression quantitative trait locus rs35197079 and decreased susceptibility of gestational diabetes mellitus in a Chinese population, Li 2062–2070

Extensor digitorum brevis muscle

Bilateral atrophy of the extensor digitorum brevis muscle might be a useful sign for diagnosing diabetic polyneuropathy in Japanese men who do not sit in the traditional “*seiza*” style, Kishimoto 398–408

Falls

Falls and fractures associated with type 2 diabetic polyneuropathy: A cross‐sectional nationwide questionnaire study, Khan 1827–1834

Fat‐free mass

Serum growth differentiation factor 11 is closely related to metabolic syndrome in a Chinese cohort, Xu 234–243

Fatty acids

Role of skeletal muscle lipids in the pathogenesis of insulin resistance of obesity and type 2 diabetes, Gilbert 1934–1941

Fatty liver

Favorable effect of sodium–glucose cotransporter 2 inhibitor, dapagliflozin, on non‐alcoholic fatty liver disease compared with pioglitazone, Cho 1272–1277
Nonalcoholic fatty liver disease is a risk factor for glucose intolerance onset in men regardless of alanine aminotransferase status, Miyake 1890–1898

Fetus

Effects of passage through the digestive tract on incretin secretion: Before and after birth, Tomotaki 970–977

Financial incentive

Screening for diabetic retinopathy with different levels of financial incentive in a randomized controlled trial, Lian 1632–1641

Fish scales

Glyoxal‐induced formation of advanced glycation end‐products in type 1 collagen decreases both its strength and flexibility *in vitro*, Kitamura 1555–1559

Fluid overload

Impact of extracellular‐to‐intracellular fluid volume ratio on albuminuria in patients with type 2 diabetes: A cross‐sectional and longitudinal cohort study, Nakajima 1202–1211

Forecasting model

Forecasting the type 2 diabetes mellitus epidemic and the role of key risk factors in Oman up to 2050: Mathematical modeling analyses, Awad 1162–1174

Fracture

Do sodium–glucose cotransporter 2 inhibitors lead to fracture risk? A pharmacovigilance real‐world study, Zhao 1400–1407

Fractures

Falls and fractures associated with type 2 diabetic polyneuropathy: A cross‐sectional nationwide questionnaire study, Khan 1827–1834

Fragmented QRS

High prevalence of fragmented QRS on electrocardiography in Japanese patients with diabetes irrespective of metabolic syndrome, Yagi 1680–1688

Frailty

Sex differences in sarcopenia and frailty among community‐dwelling Korean older adults with diabetes: The Korean Frailty and Aging Cohort Study, Kang 155–164

Fructosamine

Increased body fat mass reduces the association between fructosamine and glycated hemoglobin in obese type 2 diabetes patients, Vergès 619–624

Fuel switching

Kruppel‐like factor 15 regulates fuel switching between glucose and fatty acids in brown adipocytes, Nabatame 1144–1151

Fulminant type 1 diabetes

Onset of fulminant type 1 diabetes mellitus following hypophysitis aft er discontinuation of combined immunotherapy. A case report, Boswell, 2263–2266

Fulminant type 1 diabetes mellitus

Transition of blood glucose level in a patient with pregnancy‐associated fulminant type 1 diabetes mellitus, Ichikawa 894–896

Gas6

Growth arrest‐specific 6 modulates adiponectin expression and insulin resistance in adipose tissue, Su 485–492

Gastric emptying

Effects of glucagon‐like peptide‐1 receptor agonists on secretions of insulin and glucagon and gastric emptying in Japanese individuals with type 2 diabetes: A prospective, observational study, Kuwata 2162–2171

Genetic etiology

Clinical and genetic analysis in a Chinese cohort of children and adolescents with diabetes/persistent hyperglycemia, Ding 48–62

Gestational diabetes

Serum growth differentiation factor 15 is closely associated with metabolic abnormalities in Chinese pregnant women, Li 1501–1507
Effectiveness of antidiabetic agents for treatment of gestational diabetes: A methodological quality assessment of meta‐analyses and network meta‐analysis, Yarandi 2247–2258

Gestational diabetes mellitus

Association between neonatal birthweight and risk of maternal glucose intolerance after gestational diabetes mellitus, Li 425–433
Relationships between gut microbiota, plasma glucose and gestational diabetes mellitus, Chen 641–650
Risk factors for abnormal postpartum glycemic states in women diagnosed with gestational diabetes by the International Association of Diabetes and Pregnancy Study Groups criteria, Hung 859–868
Prevalence of non‐alcoholic fatty liver disease and factors associated with it in Indian women with a history of gestational diabetes mellitus, Kubihal 877–885
Placental maternally expressed gene 3 differentially methylated region methylation profile is associated with maternal glucose concentration and newborn birthweight, Chen 1074–1082
Risk factors and adverse maternal and perinatal outcomes for women with dichorionic twin pregnancies complicated by gestational diabetes mellitus: A retrospective cross‐sectional study, Hung 1083–1091
Diagnostic value of dysregulated microribonucleic acids in the placenta and circulating exosomes in gestational diabetes mellitus, Zhang 1490–1500
Novel association between a transient receptor potential cation channel subfamily M member 5 expression quantitative trait locus rs35197079 and decreased susceptibility of gestational diabetes mellitus in a Chinese population, Li 2062–2070
Adverse obstetric outcomes in early‐diagnosed gestational diabetes mellitus: The Japan Environment and Children’s Study, Kyozuka 2071–2079
Increasing trend in the prevalence of gestational diabetes mellitus in Taiwan, Su 2080–2088
Clinical utility of 1‐month postpartum random plasma glucose and glycated hemoglobin combined with pre‐pregnancy body mass index for detecting postpartum glucose intolerance in Japanese women with gestational diabetes, Sugiyama 2242–2246

GIP

A novel RFX6 heterozygous mutation (p.R652X) in maturity‐onset diabetes mellitus: A case report; Imaki 1914–1918

Glicentin

Pseudo‐hyperglucagonemia was observed in pancreatectomized patients when measured by glucagon sandwich enzyme‐linked immunosorbent assay, Kobayashi 286–289

Global Burden of Disease

Changing epidemiology of chronic kidney disease as a result of type 2 diabetes mellitus from 1990 to 2017: Estimates from Global Burden of Disease 2017, Li 346–356

GLP‐1

A novel RFX6 heterozygous mutation (p.R652X) in maturity‐onset diabetes mellitus: A case report; Imaki 1914–1918

GLP‐1 receptor agonist

Effects of glucagon‐like peptide‐1 receptor agonists on secretions of insulin and glucagon and gastric emptying in Japanese individuals with type 2 diabetes: A prospective, observational study, Kuwata 2162–2171

Glucagon

Pseudo‐hyperglucagonemia was observed in pancreatectomized patients when measured by glucagon sandwich enzyme‐linked immunosorbent assay, Kobayashi 286–289
Impact of glucagon response on early postprandial glucose excursions irrespective of residual β‐cell function in type 1 diabetes: A cross‐sectional study using a mixed meal tolerance test, Ito 1367–1376

Glucagon‐like peptide‐1

Effects of passage through the digestive tract on incretin secretion: Before and after birth, Tomotaki 970–977

Glucagon‐like peptide‐1 receptor agonist

Evaluating the usability and safety of the semaglutide single‐dose pen‐injectors through summative (human factors) usability testing, Klonoff 978–987
Efficacy of once‐daily glucagon‐like peptide‐1 receptor agonist lixisenatide as an add‐on treatment to basal insulin in Asian and white adults with type 2 diabetes mellitus: An individual‐level pooled analysis of phase III studies, Liu 1386–1394

Glucokinase

Glucokinase is required for high‐starch diet‐induced β‐cell mass expansion in mice, Tsuchida 1545–1554

Gluconeogenesis

Diet intake control is indispensable for the gluconeogenic response to sodium–glucose cotransporter 2 inhibition in male mice, Hashiuchi 35–47

Glucose clamp

Relation of cardiac function to insulin resistance as evaluated by hyperinsulinemic‐euglycemic clamp analysis in individuals with type 2 diabetes, Otowa‐Suematsu 2197–2202

Glucose‐dependent insulinotropic polypeptide

Effects of passage through the digestive tract on incretin secretion: Before and after birth, Tomotaki 970–977

Glucose disposal

Glucose effectiveness: Lessons from studies on insulin‐independent glucose clearance in mice, Ahrén 675–685

Glucose effectiveness

Glucose effectiveness: Lessons from studies on insulin‐independent glucose clearance in mice, Ahrén 675–685

Glucose fluctuation

Impact of daily glucose fluctuations on cardiovascular outcomes after percutaneous coronary intervention for patients with stable coronary artery disease undergoing lipid‐lowering therapy, Yamamoto 1015–1024

Glucose intolerance

Association between neonatal birthweight and risk of maternal glucose intolerance after gestational diabetes mellitus, Li 425–433

Glucose metabolism disorders

Maternal and paternal histories differentially influence risks for diabetes, insulin secretion and insulin resistance in a Chinese population, Kong 434–445

Glucose monitoring

Association between tear and blood glucose concentrations: Random intercept model adjusted with confounders in tear samples negative for occult blood, Aihara 266–276

Glucose variability

Sodium–glucose cotransporter 2 inhibitors reduce day‐to‐day glucose variability in patients with type 1 diabetes, Chiba 176–183
Impaired insulin secretion predicting unstable glycemic variability and time below range in type 2 diabetes patients regardless of glycated hemoglobin or diabetes treatment, Miya 738–746
Impact of endogenous insulin secretion on the improvement of glucose variability in Japanese patients with type 2 diabetes treated with canagliflozin plus teneligliptin, Miya 1395–1399

Glucosuria

Clinical and genetic determinants of urinary glucose excretion in patients with diabetes mellitus, Monobe 728–737

GLUT4

N4BP2L1 interacts with dynactin and contributes to GLUT4 trafficking and glucose uptake in adipocytes, Watanabe 1958–1966

Glutamate

Glutamate is an essential mediator in glutamine‐amplified insulin secretion, Han 920–930

Glutamine

Glutamate is an essential mediator in glutamine‐amplified insulin secretion, Han 920–930

Glycated hemoglobin

Association between glycemic control and cardiovascular events in older Japanese adults with diabetes mellitus: An analysis of the Japanese medical administrative database, Yokote 2036–2045

Glycemic control

Glycemic control before admission is an important determinant of prognosis in patients with coronavirus disease 2019, Liu 1064–1073

Glycemic gap

Effect of glycemic gap upon mortality in critically ill patients with diabetes, Lou 2212–2220

Glycemic variability

Defining the target value of the coefficient of variation by continuous glucose monitoring in Chinese people with diabetes, Mo 1025–1034
Metabolic and sympathovagal effects of bolus insulin glulisine versus basal insulin glargine therapy in people with type 2 diabetes: A randomized controlled study, Takeshita 1193–1201

Glycemic variability

Mean and visit‐to‐visit variability of glycated hemoglobin, and the risk of non‐alcoholic fatty liver disease, Yoo 1252–1262
Association of visit‐to‐visit glycemic variability with risk of cardiovascular diseases in high‐risk Japanese patients with type 2 diabetes: A subanalysis of the EMPATHY trial, Sato 2190–2196

Glycolysis

The PKM2 activator TEPP‐46 suppresses kidney fibrosis via inhibition of the EMT program and aberrant glycolysis associated with suppression of HIF‐1α accumulation, Liu 697–709

Glycosuria

High urinary glucose is associated with improved renal prognosis in patients with diabetes mellitus, Itano 998–1006

Glycosylated hemoglobin

Effect of glycemic gap upon mortality in critically ill patients with diabetes, Lou 2212–2220

Graves’ disease

Dipeptidyl peptidase‐4 inhibitor might exacerbate Graves’ disease: A multicenter observational case–control study, Sekizaki 1978–1982

Grit

Association between Grit Scales and adherence to regular hospital visits among Japanese patients with type 2 diabetes: Prospective observational study, Nakanishi 2259–2262

Growth differentiation factor 11

Serum growth differentiation factor 11 is closely related to metabolic syndrome in a Chinese cohort, Xu 234–243

Growth differentiation factor 15

Serum growth differentiation factor 15 is closely associated with metabolic abnormalities in Chinese pregnant women, Li 1501–1507

Gut microbiome

Relationships between gut microbiota, plasma glucose and gestational diabetes mellitus, Chen 641–650

Half marathon

Safely finishing a half marathon by an adult with type 1 diabetes using a commercially available hybrid closed‐loop system, van den Boom 450–453

Hand dexterity

Diabetic polyneuropathy and carpal tunnel syndrome together affect hand strength, tactile sensation and dexterity in diabetes patients, Zhang 2010–2018

Health‐related quality of life

Health‐related quality of life and its predictors among the type 2 diabetes population of Bangladesh: A nation‐wide cross‐sectional study, Barua 277–285
Valuing health states of people with type 2 diabetes: Analyses of the nationwide representative linked databases, Kuo 1749–1758

Health utilities

Valuing health states of people with type 2 diabetes: Analyses of the nationwide representative linked databases, Kuo 1749–1758

Heart failure

Sodium–glucose cotransporter 2 inhibitors represent a paradigm shift in the prevention of heart failure in type 2 diabetes patients Kashiwagi, 6–20
Incidence of adverse cardiovascular events in type 2 diabetes mellitus patients after initiation of glucose‐lowering agents: A population‐based community study from the Shizuoka Kokuho database, Kohsaka 1452–1461

Heart rate variability

Feasibility of combining heart rate variability and electrochemical skin conductance as screening and severity evaluation of cardiovascular autonomic neuropathy in type 2 diabetes, Lai 1671–1679

Hemoglobin A1c

Association of time in range with hemoglobin A1c, glycated albumin and 1,5‐anhydro‐d‐glucitol, Ohigashi 940–949
Blood glucose levels and bodyweight change after dapagliflozin administration, Kim 1594–1602

Heparin cofactor II

Plasma heparin cofactor II activity is inversely associated with albuminuria and its annual deterioration in patients with diabetes, Hara 2172–2182

Hepatic insulin clearance

Dipeptidyl peptidase‐4 inhibitor, anagliptin, alters hepatic insulin clearance in relation to the glycemic status in Japanese individuals with type 2 diabetes, Abe, 1805–1815

Hepatic steatosis

Serum C‐X‐C motif chemokine ligand 14 levels are associated with serum C‐peptide and fatty liver index in type 2 diabetes mellitus patients, Matsushita 1042–1049

Heritability

Genetic susceptibility, family history of diabetes and healthy lifestyle factors in relation to diabetes: A gene–environment interaction analysis in Chinese adults, Ye 2089–2098

High‐density lipoprotein cholesterol

Forty‐eight weeks of statin therapy for type 2 diabetes mellitus patients with lower extremity atherosclerotic disease: Comparison of the effects of pitavastatin and atorvastatin on lower femoral total plaque areas, Zhou 1278–1286

High‐starch diet

Glucokinase is required for high‐starch diet‐induced β‐cell mass expansion in mice, Tsuchida 1545–1554

Hip fractures

Association of hip fractures with cardiometabolic‐renal risk factors in Southern Chinese patients with type 2 diabetes – the Hong Kong Diabetes Register, Cheung 1739–1748

Hippocampus

Lower insulin secretion is associated with hippocampal and parahippocampal gyrus atrophy in elderly patients with type 2 diabetes mellitus, Adachi 1908–1913

Human factors engineering

Evaluating the usability and safety of the semaglutide single‐dose pen‐injectors through summative (human factors) usability testing, Klonoff 978–987

Hybrid closed‐loop system

Safely finishing a half marathon by an adult with type 1 diabetes using a commercially available hybrid closed‐loop system, van den Boom 450–453

Hyperaldosteronism

Impact of primary aldosteronism on renal function in patients with type 2 diabetes, Katsuragawa 217‐225

Hyperglycemia

Anthropometry did not approach adequate predictive accuracies for detecting elevated fasting plasma glucose or hemoglobin A1c levels among Chinese children aged 7–9 years, You 781–789
iGlarLixi reduces residual hyperglycemia in Japanese patients with type 2 diabetes uncontrolled on basal insulin: A post‐hoc analysis of the LixiLan JP‐L trial, Yabe 1992–2001

Hyperglycemic hyperosmotic syndrome

Clinical profiles of hyperglycemic crises: A single‐center retrospective study from Japan, Nishikawa 1359–1366

Hypertriglyceridemia

Effect of hypertriglyceridemia in dyslipidemia‐induced impaired glucose tolerance and sex differences in dietary features associated with hypertriglyceridemia among the Japanese population: The Gifu Diabetes Study, Nonoyama 771–780

Hypoglycemia

Effect of the FreeStyle Libre™ flash glucose monitoring system on glycemic control in individuals with type 2 diabetes treated with basal–bolus insulin therapy: An open label, prospective, multicenter trial in Japan, Ogawa 82–90
Relationships between time in range, glycemic variability including hypoglycemia and types of diabetes therapy in Japanese patients with type 2 diabetes mellitus: Hyogo Diabetes Hypoglycemia Cognition Complications study, Kuroda 244–253
Combination of alcohol and glucose consumption as a risk to induce reactive hypoglycemia, Oba‐Yamamoto 651–657
Impaired insulin secretion predicting unstable glycemic variability and time below range in type 2 diabetes patients regardless of glycated hemoglobin or diabetes treatment, Miya 738–746

Hypophosphatemia

Fractional excretion of tumor necrosis factor receptor 1 and 2 in patients with type 2 diabetes and normal renal function, Gohda 382–389

Hypophysitis

Onset of fulminant type 1 diabetes mellitus following hypophysitis aft er discontinuation of combined immunotherapy. A case report, Boswell, 2263–2266

Immunotherapy

Onset of fulminant type 1 diabetes mellitus following hypophysitis aft er discontinuation of combined immunotherapy. A case report, Boswell, 2263–2266

Impaired fasting glucose

Association between plasma irisin in pregnancy and postpartum glucose levels among Chinese women: A cohort study, Tang 1723–1731

Impaired glucose tolerance

Ingestion of an exogenous ketone monoester improves the glycemic response during oral glucose tolerance test in individuals with impaired glucose tolerance: A cross‐over randomized trial, Nakagata 756–762
Effect of hypertriglyceridemia in dyslipidemia‐induced impaired glucose tolerance and sex differences in dietary features associated with hypertriglyceridemia among the Japanese population: The Gifu Diabetes Study, Nonoyama 771–780

Incentive reimbursement

Synergy between the pay‐for‐performance scheme and better physician–patient relationship might reduce the risk of retinopathy in patients with type 2 diabetes, Chiou 819–827

Incident diabetes

Low‐density lipoprotein cholesterol : high‐density lipoprotein cholesterol ratio is associated with incident diabetes in Chinese adults: A retrospective cohort study, Wei 91–98

Inflammation

Human SHC‐transforming protein 1 and its isoforms p66shc: A novel marker for prediabetes, Jelinek 1881–1889

Influenza

Association between influenza and the incidence rate of new‐onset type 1 diabetes in Japan, Nishioka 1797–1804

Inpatient glycemic management

Electronic dashboard‐based remote glycemic management program reduces length of stay and readmission rate among hospitalized adults, Sheen 1697–1707

Islet hormones

Effects of glucagon‐like peptide‐1 receptor agonists on secretions of insulin and glucagon and gastric emptying in Japanese individuals with type 2 diabetes: A prospective, observational study, Kuwata 2162–2171

Insulin

Risk of early mortality and cardiovascular disease according to the presence of recently diagnosed diabetes and requirement for insulin treatment: A nationwide study, Lee 1855–1863

Insulin degludec

Comparative clinical outcomes of insulin degludec and insulin glargine 300 U/mL after switching from other basal insulins in real‐world patients with type 1 and type 2 diabetes, Oya 1983–1991

Insulin degludec/liraglutide

Efficacy and safety of the fixed‐ratio combination of insulin degludec and liraglutide by baseline glycated hemoglobin, body mass index and age in Japanese individuals with type 2 diabetes: A subgroup analysis of two phase III trials, Komatsu 1610–1618

Insulin glargine 300 U/mL

Comparative clinical outcomes of insulin degludec and insulin glargine 300 U/mL after switching from other basal insulins in real‐world patients with type 1 and type 2 diabetes, Oya 1983–1991

Insulin persistence

Persons with type 2 diabetes and high insulin persistence were associated with a lower risk of mortality: A nationwide retrospective cohort study, Yen 146–154

Insulin resistance

Loss of metabolic flexibility as a result of overexpression of pyruvate dehydrogenase kinases in muscle, liver and the immune system: Therapeutic targets in metabolic diseases Jeon, 21‐31
Association between the triglyceride–glucose index and diabetic nephropathy in patients with type 2 diabetes: A cross‐sectional study, Liu 557–565
Potassium supplementation blunts the effects of high salt intake on serum retinol‐binding protein 4 levels in healthy individuals, Liu 658–663
Serum C‐X‐C motif chemokine ligand 14 levels are associated with serum C‐peptide and fatty liver index in type 2 diabetes mellitus patients, Matsushita 1042–1049
Insulin resistance is independently associated with cardiovascular autonomic neuropathy in type 2 diabetes, Liu 1651–1662
Novel *PIK3R1* mutation of SHORT syndrome: A case report with a 6‐month follow up, Yin 1919–1922
Insulin resistance limits corneal nerve regeneration in patients with type 2 diabetes undergoing intensive glycemic control, Ponirakis 2002–2009
Relation of cardiac function to insulin resistance as evaluated by hyperinsulinemic‐euglycemic clamp analysis in individuals with type 2 diabetes, Otowa‐Suematsu 2197–2202

Insulin secretion

Impaired insulin secretion predicting unstable glycemic variability and time below range in type 2 diabetes patients regardless of glycated hemoglobin or diabetes treatment, Miya 738–746
Ingestion of an exogenous ketone monoester improves the glycemic response during oral glucose tolerance test in individuals with impaired glucose tolerance: A cross‐over randomized trial, Nakagata 756–762
Moderate alcohol consumption is associated with impaired insulin secretion and fasting glucose in non‐obese non‐diabetic men, Miyagi 869–876
Glutamate is an essential mediator in glutamine‐amplified insulin secretion, Han 920–930
Lower insulin secretion is associated with hippocampal and parahippocampal gyrus atrophy in elderly patients with type 2 diabetes mellitus, Adachi 1908–1913
Using recombinase‐mediated cassette exchange to engineer MIN6 insulin‐secreting cells based on a newly identified safe harbor locus, Furukawa 2129–2140

Insulin therapy

Metabolic and sympathovagal effects of bolus insulin glulisine versus basal insulin glargine therapy in people with type 2 diabetes: A randomized controlled study, Takeshita 1193–1201

Intensive insulin treatment

Type 1 diabetes management and outcomes: A multicenter study in Thailand, Dejkhamron 516–526

Intermittently scanned continuous glucose monitoring

Effect of the FreeStyle Libre™ flash glucose monitoring system on glycemic control in individuals with type 2 diabetes treated with basal–bolus insulin therapy: An open label, prospective, multicenter trial in Japan, Ogawa 82–90

International Association of Diabetes and Pregnancy Study Groups criteria

Prevalence of non‐alcoholic fatty liver disease and factors associated with it in Indian women with a history of gestational diabetes mellitus, Kubihal 877–885

Intraoperative euglycemic ketoacidosis

Early detection of euglycemic ketoacidosis during thoracic surgery associated with empagliflozin in a patient with type 2 diabetes: A case report, Kitahara 664–667

Irisin

Irisin plays an important role in the outcomes of newly diagnosed prediabetes in adults in Guiyang, China, He 747–755
Association between plasma irisin in pregnancy and postpartum glucose levels among Chinese women: A cohort study, Tang 1723–1731

Japan

Perceptions, attitudes and barriers to obesity management: Japanese data from the ACTION‐IO study, Iwabu 845–858
Efficacy and safety of the fixed‐ratio combination of insulin degludec and liraglutide by baseline glycated hemoglobin, body mass index and age in Japanese individuals with type 2 diabetes: A subgroup analysis of two phase III trials, Komatsu 1610–1618
iGlarLixi reduces residual hyperglycemia in Japanese patients with type 2 diabetes uncontrolled on basal insulin: A post‐hoc analysis of the LixiLan JP‐L trial, Yabe 1992–2001

Japan Association of Diabetes Education and Care

Effects of physician’s diabetes self‐management education using Japan Association of Diabetes Education and Care Diabetes Education Card System Program and a self‐monitoring of blood glucose readings analyzer in individuals with type 2 diabetes: An exploratory, open‐labeled, prospective randomized clinical trial, Tanaka 2221–2231

Japanese adults

Clinical inertia in patients with type 2 diabetes treated with oral antidiabetic drugs: Results from a Japanese cohort study (JDDM53), Maegawa 374–381

Japanese meal

Frequency of consumption of balanced meals, bodyweight gain and incident risk of glucose intolerance in Japanese men and women: A cohort study, Sakurai 763–770

JDI Updates

Advancements in transplantation therapy for diabetes: Pancreas, islet and stem cell, Nakamura 143–145
In search of the optimal management strategy for non‐alcoholic fatty liver disease in type 2 diabetes patients, Lee 482–484
Recent evidence in the etiology and treatment for diabetic kidney disease, Shikata 694–696
Two types of fulminant type 1 diabetes mellitus: Immune checkpoint inhibitor‐related and conventional, Imagawa 917–919
New prospects for incretin‐related drugs in the treatment of type 2 diabetes, Kimura 1141–1143
Update in the epidemiology, risk factors, screening, and treatment of diabetic retinopathy, Lin 1322–1325
Blood glucose control: Where are we?, Lee 1762–1764
Recent updates and future perspectives on gestational diabetes mellitus: An important public health challenge, Chu 1944–1947

Ketone

Ingestion of an exogenous ketone monoester improves the glycemic response during oral glucose tolerance test in individuals with impaired glucose tolerance: A cross‐over randomized trial, Nakagata 756–762

Kidney transplantation

Trends in the effects of pre‐transplant diabetes on mortality and cardiovascular events after kidney transplantation, Jeon 811–818

Kruppel‐like factor 15

Kruppel‐like factor 15 regulates fuel switching between glucose and fatty acids in brown adipocytes, Nabatame 1144–1151

Laparoscopic sleeve gastrectomy

Metabolic alterations in plasma after laparoscopic sleeve gastrectomy, Yoshida 123–129

Left ventricular cardiac dysfunction

Relation of cardiac function to insulin resistance as evaluated by hyperinsulinemic‐euglycemic clamp analysis in individuals with type 2 diabetes, Otowa‐Suematsu 2197–2202

Length of stay

Electronic dashboard‐based remote glycemic management program reduces length of stay and readmission rate among hospitalized adults, Sheen 1697–1707

Léri–Weill dyschondrosteosis

Metabolic syndrome coexists with adult Léri–Weill dyschondrosteosis: A case report, Wang 446–449

Letter to the Editor

Protein ingestion can significantly affect glucagon secretion along with blood urea nitrogen alteration in type 1 diabetes, Suzuki 293–294
Focus on nerve fiber type: A diagnostic strategy for diabetic polyneuropathy, Fustes 459–460
Response to ‘Focus on nerve fiber type: A diagnostic strategy for diabetic polyneuropathy’, Sasaki 461
Relationship between soluble transferrin receptor and type 2 diabetes mellitus: A meta‐analysis, Fernández‐Cao 1118–1119
Response to “Relationship between soluble transferrin receptor and type 2 diabetes mellitus: A meta‐analysis”, Liu 1120–1121
Effectiveness of remote continuous glucose monitoring on adverse outcomes among patients with diabetes complicated with COVID‐19, Shen 1923–1924
Acute and extremely severe necrotic esophagitis accompanied by hyperglycemic hyperosmolar syndrome in a subject with type 2 diabetes mellitus, Iwamoto 1925–1926
Multiple skin ulceration and itch–scratch cycle in a diabetic patient, Murao 2102–2103
New strategy for diagnosing abnormal glucose tolerance before 24 gestational weeks during the coronavirus disease 2019 pandemic, Kasuga 2104‐2105

Leukocyte telomere length

Reduced leukocyte telomere lengths and sirtuin 1 gene expression in long‐term survivors of type 1 diabetes: A Dialong substudy, Opstad 1183–1192

Lifestyle

Genetic susceptibility, family history of diabetes and healthy lifestyle factors in relation to diabetes: A gene–environment interaction analysis in Chinese adults, Ye 2089–2098

Liver fibrosis

Liver fibrosis is independently associated with diabetic peripheral neuropathy in type 2 diabetes mellitus, Huang 2019–2027

Liver steatosis

Liver fibrosis is independently associated with diabetic peripheral neuropathy in type 2 diabetes mellitus, Huang 2019–2027

Liver transaminases

Plasma xanthine oxidoreductase activity in Japanese patients with type 2 diabetes across hospitalized treatment, Kawachi 1512–1520

Lixisenatide

Efficacy of once‐daily glucagon‐like peptide‐1 receptor agonist lixisenatide as an add‐on treatment to basal insulin in Asian and white adults with type 2 diabetes mellitus: An individual‐level pooled analysis of phase III studies, Liu 1386–1394

Long‐term prognosis

Admission hyperglycemia as an independent predictor of long‐term prognosis in acute myocardial infarction patients without diabetes: A retrospective study, Cui 1244–1251

Low‐density lipoprotein cholesterol : high‐density lipoprotein cholesterol ratio

Low‐density lipoprotein cholesterol : high‐density lipoprotein cholesterol ratio is associated with incident diabetes in Chinese adults: A retrospective cohort study, Wei 91–98

Lower extremity atherosclerotic disease

Forty‐eight weeks of statin therapy for type 2 diabetes mellitus patients with lower extremity atherosclerotic disease: Comparison of the effects of pitavastatin and atorvastatin on lower femoral total plaque areas, Zhou 1278–1286

Lymphocyte

Vitamin D affects the neutrophil‐to‐lymphocyte ratio in patients with type 2 diabetes mellitus, Wang 254–265

Maternally expressed gene 3 differentially methylated region

Placental maternally expressed gene 3 differentially methylated region methylation profile is associated with maternal glucose concentration and newborn birthweight, Chen 1074–1082

Mathematical modeling

Glucose effectiveness: Lessons from studies on insulin‐independent glucose clearance in mice, Ahrén 675–685

Maturity‐onset diabetes of the young

Recognition of maturity‐onset diabetes of the young in China, Liang 501–509

Meal tolerance test

Impact of glucagon response on early postprandial glucose excursions irrespective of residual β‐cell function in type 1 diabetes: A cross‐sectional study using a mixed meal tolerance test, Ito 1367–1376
Dipeptidyl peptidase‐4 inhibitor, anagliptin, alters hepatic insulin clearance in relation to the glycemic status in Japanese individuals with type 2 diabetes, Abe, 1805–1815

Mean corpuscular volume

Association of mean corpuscular volume with sarcopenia and visceral obesity in individuals without anemia, Tanaka 1287–1292

Medical history taking

Maternal and paternal histories differentially influence risks for diabetes, insulin secretion and insulin resistance in a Chinese population, Kong 434–445

Mediterranean diet

East Asian diet‐mimicking diet plan based on the Mediterranean diet and the Dietary Approaches to Stop Hypertension diet in adults with type 2 diabetes: A randomized controlled trial, Jin 357–364

Melatonin

Associations between urinary 6‐sulfatoxymelatonin excretion and diabetic vascular complications or arteriosclerosis in patients with type 2 diabetes, Tanaka 601–609

Meta‐analysis

Risk factors for new‐onset diabetes mellitus after kidney transplantation: A systematic review and meta‐analysis, Xia 109–122
Effects of mindfulness‐based intervention on glycemic control and psychological outcomes in people with diabetes: A systematic review and meta‐analysis, Ni 1092–1103

Metabolic abnormalities

Serum growth differentiation factor 15 is closely associated with metabolic abnormalities in Chinese pregnant women, Li 1501–1507

Metabolically healthy

Sex differences in the association between fatty liver and type 2 diabetes incidence in non‐obese Japanese: A retrospective cohort study, Narisada 1480–1489

Metabolic disorders

Serum growth differentiation factor 11 is closely related to metabolic syndrome in a Chinese cohort, Xu 234–243

Metabolites

Metabolic alterations in plasma after laparoscopic sleeve gastrectomy, Yoshida 123–129

Metabolomics

Observational study of diabetes inpatients with gas chromatography/mass spectrometry‐based non‐target metabolomic analysis, Taya 2232–2241

Metformin

Effects of ipragliflozin versus metformin in combination with sitagliptin on bone and muscle in Japanese patients with type 2 diabetes mellitus: Subanalysis of a prospective, randomized, controlled study (PRIME‐V study), Koshizaka 200–206
Metformin as a potential protective therapy against tuberculosis in patients with diabetes mellitus: A retrospective cohort study in a single teaching hospital, Fu 1603–1609

Microangiopathy

Islet microangiopathy and augmented β‐cell loss in Japanese non‐obese type 2 diabetes patients who died of acute myocardial infarction, Takahashi 2149–2161

Microcapsules

Microencapsulation of cells and molecular therapy of type 1 diabetes mellitus: The actual state and future perspectives between promise and progress, Basta 301–309

Microribonucleic acid

Correlation analysis of microribonucleic acid‐155 and microribonucleic acid‐29 with type 2 diabetes mellitus, and the prediction and verification of target genes, Zhu 165–175

Micro‐ribonucleic acid‐23a‐3p

Micro‐ribonucleic acid‐23a‐3p prevents the onset of type 2 diabetes mellitus by suppressing the activation of nucleotide‐binding oligomerization‐like receptor family pyrin domain containing 3 inflammatory bodies‐caused pyroptosis through negatively regulating NIMA‐related kinase 7, Chang 334–345

Mindfulness‐based intervention

Effects of mindfulness‐based intervention on glycemic control and psychological outcomes in people with diabetes: A systematic review and meta‐analysis, Ni 1092–1103

Mitochondria

Role of skeletal muscle lipids in the pathogenesis of insulin resistance of obesity and type 2 diabetes, Gilbert 1934–1941

Mortality

Trends in the effects of pre‐transplant diabetes on mortality and cardiovascular events after kidney transplantation, Jeon 811–818
All‐cause and cardiovascular disease mortality in underweight patients with diabetic nephropathy: BioBank Japan cohort, Yokomichi 1425–1429
Risk of early mortality and cardiovascular disease according to the presence of recently diagnosed diabetes and requirement for insulin treatment: A nationwide study, Lee 1855–1863

Multiple daily insulin injection

Type 1 diabetes management and outcomes: A multicenter study in Thailand, Dejkhamron 516–526

Muscle strength

Loss of lower extremity muscle strength based on diabetic polyneuropathy in older patients with type 2 diabetes: Multicenter Survey of the Isometric Lower Extremity Strength in Type 2 Diabetes: Phase 2 study, Nomura 390–397

N4BP2L1

N4BP2L1 interacts with dynactin and contributes to GLUT4 trafficking and glucose uptake in adipocytes, Watanabe 1958–1966

N6‐methyladenosine

Epigenetic regulation of N6‐methyladenosine modifications in obesity, Sun 1306–1315

Na+/Ca2+ exchanger

Calcium signaling in endocardial and epicardial ventricular myocytes from streptozotocin‐induced diabetic rats, Al Kury 493–500

Nailfold capillaroscopy

Association of crossing capillaries in the finger nailfold with diabetic retinopathy in type 2 diabetes mellitus, Shikama 1007–1014

National Health Insurance Research Database

Increasing trend in the prevalence of gestational diabetes mellitus in Taiwan, Su 2080–2088

Nerve conduction study

Association of platelet count and plateletcrit with nerve conduction function and peripheral neuropathy in patients with type 2 diabetes mellitus, Qian 1835–1844

Network meta‐analysis

Effectiveness of antidiabetic agents for treatment of gestational diabetes: A methodological quality assessment of meta‐analyses and network meta‐analysis, Yarandi 2247–2258

Neuroretina

Alterations of retinal thickness measured by optical coherence tomography correlate with neurophysiological measures in diabetic polyneuropathy, Yamada 1430–1441

Neutrophil

Vitamin D affects the neutrophil‐to‐lymphocyte ratio in patients with type 2 diabetes mellitus, Wang 254–265

New‐onset diabetes mellitus after kidney transplantation

Risk factors for new‐onset diabetes mellitus after kidney transplantation: A systematic review and meta‐analysis, Xia 109–122

Next‐generation sequencing

Clinical and genetic analysis in a Chinese cohort of children and adolescents with diabetes/persistent hyperglycemia, Ding 48–62

Nicorandil

Nicorandil decreases oxidative stress in slow‐ and fast‐twitch muscle fibers of diabetic rats by improving the glutathione system functioning, Sánchez‐Duarte 1152–1161

Nocturnal asymptomatic hypoglycemia

Is it possible to predict the onset of nocturnal asymptomatic hypoglycemia in patients with type 1 diabetes receiving insulin degludec? Potential role of previous day and next morning glucose values, Takahashi 365–373

NOD‐LRR‐ and pyrin domain‐containing protein 3

Micro‐ribonucleic acid‐23a‐3p prevents the onset of type 2 diabetes mellitus by suppressing the activation of nucleotide‐binding oligomerization‐like receptor family pyrin domain containing 3 inflammatory bodies‐caused pyroptosis through negatively regulating NIMA‐related kinase 7, Chang 334–345

Non‐alcoholic fatty liver disease

Prevalence of non‐alcoholic fatty liver disease and factors associated with it in Indian women with a history of gestational diabetes mellitus, Kubihal 877–885
Mean and visit‐to‐visit variability of glycated hemoglobin, and the risk of non‐alcoholic fatty liver disease, Yoo 1252–1262
Decreased plasma n6 : n3 polyunsaturated fatty acids ratio interacting with high C‐peptide promotes non‐alcoholic fatty liver disease in type 2 diabetes patients, Luo 1263–1271
Association of non‐alcoholic fatty liver disease with diabetic retinopathy in type 2 diabetic patients: A meta‐analysis of observational studies, Song 1471–1479

Non‐alcoholic fatty liver disease fibrosis score

Association of the Non‐Alcoholic Fatty Liver Disease Fibrosis Score with subclinical myocardial remodeling in patients with type 2 diabetes: A cross‐sectional study in China, Fan 1035–1041

Non‐communicable disease

Forecasting the type 2 diabetes mellitus epidemic and the role of key risk factors in Oman up to 2050: Mathematical modeling analyses, Awad 1162–1174

Non‐fasting hypertriglyceridemia

Thresholds for postprandial hyperglycemia and hypertriglyceridemia associated with increased mortality risk in type 2 diabetes patients: A real‐world longitudinal study, Takao 886–893

Non‐invasive

Association between tear and blood glucose concentrations: Random intercept model adjusted with confounders in tear samples negative for occult blood, Aihara 266–276

Normal alanine aminotransferase

Nonalcoholic fatty liver disease is a risk factor for glucose intolerance onset in men regardless of alanine aminotransferase status, Miyake 1890–1898

Nutritional epidemiology

Frequency of consumption of balanced meals, bodyweight gain and incident risk of glucose intolerance in Japanese men and women: A cohort study, Sakurai 763–770

Nutrition guidance

Association between nutritional guidance or ophthalmological examination and discontinuation of physician visits in patients with newly diagnosed diabetes: A retrospective cohort study using a nationwide database, Akira Okada 1619–1631

Obese

Increased body fat mass reduces the association between fructosamine and glycated hemoglobin in obese type 2 diabetes patients, Vergès 619–624

Obesity

Metabolic alterations in plasma after laparoscopic sleeve gastrectomy, Yoshida 123–129
Correlation analysis of microribonucleic acid‐155 and microribonucleic acid‐29 with type 2 diabetes mellitus, and the prediction and verification of target genes, Zhu 165–175
Perceptions, attitudes and barriers to obesity management: Japanese data from the ACTION‐IO study, Iwabu 845–858
Potential biomarker in serum for predicting susceptibility to type 2 diabetes mellitus: Free fatty acid 22:6, Ma 950–962
Epigenetic regulation of N6‐methyladenosine modifications in obesity, Sun 1306–1315
Association of protein tyrosine phosphatase 1B gene polymorphism with the effects of weight reduction therapy on bodyweight and glycolipid profiles in obese patients, Yamakage 1462–1470

Obesity management

Perceptions, attitudes and barriers to obesity management: Japanese data from the ACTION‐IO study, Iwabu 845–858

Obituary

Obituary of Dr. Susumu Seino, Inagaki 2106

Older diabetes patients

Association between glycemic control and cardiovascular events in older Japanese adults with diabetes mellitus: An analysis of the Japanese medical administrative database, Yokote 2036–2045

Older patients

Loss of lower extremity muscle strength based on diabetic polyneuropathy in older patients with type 2 diabetes: Multicenter Survey of the Isometric Lower Extremity Strength in Type 2 Diabetes: Phase 2 study, Nomura 390–397

O‐linked N‐acetylglucosamine modification

Role of O‐linked N‐acetylglucosamine in the homeostasis of metabolic organs, and its potential links with diabetes and its complications, Morino 130–136

Oral glucose tolerance test

Relationships between gut microbiota, plasma glucose and gestational diabetes mellitus, Chen 641–650
Combination of alcohol and glucose consumption as a risk to induce reactive hypoglycemia, Oba‐Yamamoto 651–657

Osteoporosis

Beneficial effects of switching to denosumab from bisphosphonates or selective estrogen receptor modulators in postmenopausal women with type 2 diabetes and osteopenia/osteoporosis, Miyoshi 1293–1300

Oxidative stress

Nicorandil decreases oxidative stress in slow‐ and fast‐twitch muscle fibers of diabetic rats by improving the glutathione system functioning, Sánchez‐Duarte 1152–1161
β‐Cell failure in diabetes: Common susceptibility and mechanisms shared between type 1 and type 2 diabetes, Ikegami 1526–1539
Human SHC‐transforming protein 1 and its isoforms p66shc: A novel marker for prediabetes, Jelinek 1881–1889
Bilirubin is inversely related to diabetic peripheral neuropathy assessed by sural nerve conduction study, Abe 2028–2035

Painful diabetic neuropathy

Association of time in range, as assessed by continuous glucose monitoring, with painful diabetic polyneuropathy, Yang 828–836
Painful diabetic neuropathy is associated with increased nerve regeneration in patients with type 2 diabetes undergoing intensive glycemic control, Ponirakis 1642–1650

Painful neuropathy

Prevalence and risk factors for diabetic neuropathy and painful diabetic neuropathy in primary and secondary healthcare in Qatar, Ponirakis 592–600

Pancreas aging

Islet β‐cells physiological difference study of old and young mice based on single‐cell transcriptomics, Zheng 1775–1783

Pancreatectomy

Pseudo‐hyperglucagonemia was observed in pancreatectomized patients when measured by glucagon sandwich enzyme‐linked immunosorbent assay, Kobayashi 286–289

Pancreatic β‐cells

Inverse correlation between serum high‐molecular‐weight adiponectin and proinsulin level in a Japanese population: The Dynamics of Lifestyle and Neighborhood Community on Health Study, Nakamura 63–66

Pancreatic islet development

Investigating the role of *dachshund b* in the development of the pancreatic islet in zebrafish, Yang 710–727

Parathyroid hormone

Normal parathyroid hormone and non‐proliferative diabetic retinopathy in patients with type 2 diabetes, Sun 1220–1227

Pathogenic genes

Recognition of maturity‐onset diabetes of the young in China, Liang 501–509

Pediatric diabetes

Epidemiology, genetic landscape and classifi cation of childhood diabetes mellitus in the State of Qatarm Haris 2141–2148

Pen‐injector

Evaluating the usability and safety of the semaglutide single‐dose pen‐injectors through summative (human factors) usability testing, Klonoff 978–987

Pentosidine

Elevated serum pentosidine is independently associated with the high prevalence of sarcopenia in Chinese middle‐aged and elderly men with type 2 diabetes mellitus, Zhang 2054–2061

Periodontal disease

Epigenetic changes caused by diabetes and their potential role in the development of periodontitis, Li 1326–1335

Perinatal outcomes

Risk factors and adverse maternal and perinatal outcomes for women with dichorionic twin pregnancies complicated by gestational diabetes mellitus: A retrospective cross‐sectional study, Hung 1083–1091

Peripheral neuropathy

Diabetes coexistent with Charcot–Marie–Tooth disease presenting as a recurrent foot ulcer misdiagnosed as diabetic foot: A case report, Yan 2099–2101

Pharmacotherapy

Current concepts in the management of diabetic polyneuropathy, Ziegler 464–475

Pharmacovigilance

Do sodium–glucose cotransporter 2 inhibitors lead to fracture risk? A pharmacovigilance real‐world study, Zhao 1400–1407

Phosphate

Fractional excretion of tumor necrosis factor receptor 1 and 2 in patients with type 2 diabetes and normal renal function, Gohda 382–389

Physical activity

Predictors of the maximal oxygen consumption in adult patients with type 1 diabetes treated with personal insulin pumps, Matejko 1377–1385
Lifestyle changes as a result of COVID‐19 containment measures: Bodyweight and glycemic control in patients with diabetes in the Japanese declaration of a state of emergency, Tanaka 1718–1722

PIK3R1

Novel *PIK3R1* mutation of SHORT syndrome: A case report with a 6‐month follow up, Yin 1919–1922

Plant‐based diet

Association of plant‐based diet and type 2 diabetes mellitus in Chinese rural adults: The Henan Rural Cohort Study, Yang 1569–1576

Platelet indices

Association of platelet count and plateletcrit with nerve conduction function and peripheral neuropathy in patients with type 2 diabetes mellitus, Qian 1835–1844

Point‐of‐care testing

Point‐of‐care nerve conduction device predicts the severity of diabetic polyneuropathy: A quantitative, but easy‐to‐use, prediction model, Kamiya 583–591
Neuroretinal dysfunction revealed by a flicker electroretinogram correlated with peripheral nerve dysfunction and parameters of atherosclerosis in patients with diabetes, Kawai 1236–1243

Polyunsaturated fatty acids

Decreased plasma n6 : n3 polyunsaturated fatty acids ratio interacting with high C‐peptide promotes non‐alcoholic fatty liver disease in type 2 diabetes patients, Luo 1263–1271

Pooled analysis

Efficacy of once‐daily glucagon‐like peptide‐1 receptor agonist lixisenatide as an add‐on treatment to basal insulin in Asian and white adults with type 2 diabetes mellitus: An individual‐level pooled analysis of phase III studies, Liu 1386–1394

Post‐marketing surveillance

Safety and effectiveness of tofogliflozin in Japanese patients with type 2 diabetes mellitus treated in real‐world clinical practice: Results of a 36‐month post‐marketing surveillance study (J‐STEP/LT), Utsunomiya 184–199

Postpartum

Risk factors for abnormal postpartum glycemic states in women diagnosed with gestational diabetes by the International Association of Diabetes and Pregnancy Study Groups criteria, Hung 859–868

Postpartum glucose intolerance

Clinical utility of 1‐month postpartum random plasma glucose and glycated hemoglobin combined with pre‐pregnancy body mass index for detecting postpartum glucose intolerance in Japanese women with gestational diabetes, Sugiyama 2242–2246

Postpartum women

Association between plasma irisin in pregnancy and postpartum glucose levels among Chinese women: A cohort study, Tang 1723–1731

Post‐prandial blood glucose

Improved time in range and postprandial hyperglycemia with canagliflozin in combination with teneligliptin: Secondary analyses of the CALMER study, Cho 1417–1424

Postprandial hyperglycemia

Thresholds for postprandial hyperglycemia and hypertriglyceridemia associated with increased mortality risk in type 2 diabetes patients: A real‐world longitudinal study, Takao 886–893

Postprandial hypotension

Asymptomatic postprandial hypotension in patients with diabetes: The KAMOGAWA‐HBP study, Kitae 837–844

Post‐translational modification

Role of O‐linked N‐acetylglucosamine in the homeostasis of metabolic organs, and its potential links with diabetes and its complications, Morino 130–136

Poverty

Non‐financial social determinants of diabetes among public assistance recipients in Japan: A cohort study, Nishioka 1104–1111

PPARγ

SIRT7 regulates lipogenesis in adipocytes through deacetylation of PPARγ2, Akter 1765–1774

Practical screening method

Bilateral atrophy of the extensor digitorum brevis muscle might be a useful sign for diagnosing diabetic polyneuropathy in Japanese men who do not sit in the traditional “*seiza*” style, Kishimoto 398–408

Prediabetes

Prediabetes and long‐term outcomes in patients with three‐vessel coronary artery disease: A large single‐center cohort study, Yuan 409–416
Irisin plays an important role in the outcomes of newly diagnosed prediabetes in adults in Guiyang, China, He 747–755
Simple risk score to screen for prediabetes: A cross‐sectional study from the Qatar Biobank cohort, Abbas 988–997
Human SHC‐transforming protein 1 and its isoforms p66shc: A novel marker for prediabetes, Jelinek 1881–1889

Pregnancy

Transition of blood glucose level in a patient with pregnancy‐associated fulminant type 1 diabetes mellitus, Ichikawa 894–896

Preoperative fundus examination

Preoperative fundus examination in patients with diabetes scheduled for surgery, Watanabe 1508–1511

Prevalence

Increasing trend in the prevalence of gestational diabetes mellitus in Taiwan, Su 2080–2088

Prognosis

Glycemic control before admission is an important determinant of prognosis in patients with coronavirus disease 2019, Liu 1064–1073

Prognostic factors

Epidemiological characteristics of diabetic kidney disease in Taiwan, Wang 2112–2123

Progression of diabetes mellitus

Mediation effect of the duration of diabetes mellitus on the decrease in bioimpedance phase angles in ethnically Korean people: A multicenter clinical study, Jun 790–802

Proinsulin

Inverse correlation between serum high‐molecular‐weight adiponectin and proinsulin level in a Japanese population: The Dynamics of Lifestyle and Neighborhood Community on Health Study, Nakamura 63–66

Prolactinoma

Rapid and dramatic glucose‐lowering effect of bromocriptine in an inadequately controlled type 2 diabetes patient with prolactinoma, Igata 668–671

Prospective cohort study

Midlife and late‐life diabetes and sarcopenia in a general older Japanese population: The Hisayama Study, Nakamura 1899–1907

Protease‐activated receptors

Plasma heparin cofactor II activity is inversely associated with albuminuria and its annual deterioration in patients with diabetes, Hara 2172–2182

Protein tyrosine phosphatase 1B

Association of protein tyrosine phosphatase 1B gene polymorphism with the effects of weight reduction therapy on bodyweight and glycolipid profiles in obese patients, Yamakage 1462–1470

Public assistance

Non‐financial social determinants of diabetes among public assistance recipients in Japan: A cohort study, Nishioka 1104–1111

Pyogenic psoas abscess

Pyogenic psoas abscess on the dorsal side, and bacterial meningitis and spinal epidural abscess on the ventral side, both of which were induced by spontaneous discitis in a patient with diabetes mellitus: A case report, Horiya 1301–1305

Pyruvate dehydrogenase kinase

Loss of metabolic flexibility as a result of overexpression of pyruvate dehydrogenase kinases in muscle, liver and the immune system: Therapeutic targets in metabolic diseases Jeon, 21‐31

Qatar Biobank

Simple risk score to screen for prediabetes: A cross‐sectional study from the Qatar Biobank cohort, Abbas 988–997

Quality of life

Simplification of complex insulin regimens using canagliflozin or liraglutide in patients with well‐controlled type 2 diabetes: A 24‐week randomized controlled trial, Ando 1816–1826

Ramadan

Readmission

Real‐world clinical setting

Comparative clinical outcomes of insulin degludec and insulin glargine 300 U/mL after switching from other basal insulins in real‐world patients with type 1 and type 2 diabetes, Oya 1983–1991

Recombination mediated cassette exchange

Using recombinase‐mediated cassette exchange to engineer MIN6 insulin‐secreting cells based on a newly identified safe harbor locus, Furukawa 2129–2140

Renal insufficiency

Impact of primary aldosteronism on renal function in patients with type 2 diabetes, Katsuragawa 217‐225
High urinary glucose is associated with improved renal prognosis in patients with diabetes mellitus, Itano 998–1006

Retinal vessels

Imaging the eye and its relevance to diabetes care, Quinn 897–908

Retinol‐binding protein 4

Potassium supplementation blunts the effects of high salt intake on serum retinol‐binding protein 4 levels in healthy individuals, Liu 658–663

Retinopathy complication

Serum progesterone and retinopathy in male patients with type 2 diabetes: A cross‐sectional study, Sun 1228–1235

RFX6

A novel RFX6 heterozygous mutation (p.R652X) in maturity‐onset diabetes mellitus: A case report; Imaki 1914–1918

Risk factor

Forecasting the type 2 diabetes mellitus epidemic and the role of key risk factors in Oman up to 2050: Mathematical modeling analyses, Awad 1162–1174

Risk factors

Risk factors for new‐onset diabetes mellitus after kidney transplantation: A systematic review and meta‐analysis, Xia 109–122
Cardiovascular events and mortality in people with and without type 2 diabetes: An observational study in a contemporary multi‐ethnic population, Coles 1175–1182
One‐year estimated glomerular filtration rate decline as a risk factor of cardiovascular and renal end‐points in high‐risk Japanese patients, Meguro 1212–1219
Epidemiological characteristics of diabetic kidney disease in Taiwan, Wang 2112–2123

Risk model

Development of a clinical risk score for incident diabetes: A 10‐year prospective cohort study, Oh 610–618

Risk score

Simple risk score to screen for prediabetes: A cross‐sectional study from the Qatar Biobank cohort, Abbas 988–997

Rural population

Association of plant‐based diet and type 2 diabetes mellitus in Chinese rural adults: The Henan Rural Cohort Study, Yang 1569–1576

Sarcopenia

Sex differences in sarcopenia and frailty among community‐dwelling Korean older adults with diabetes: The Korean Frailty and Aging Cohort Study, Kang 155–164
Significance of body mass index for diagnosing sarcopenia is equivalent to slow gait speed in Japanese individuals with type 2 diabetes: Cross‐sectional study using outpatient clinical data, Nakanishi 417–424
Associations between sarcopenia and white matter alterations in older adults with diabetes mellitus: A diffusion tensor imaging study, Tamura 633–640
High prevalence and clinical impact of dynapenia and sarcopenia in Japanese patients with type 1 and type 2 diabetes: Findings from the Impact of Diabetes Mellitus on Dynapenia study, Mori 1050–1059
Midlife and late‐life diabetes and sarcopenia in a general older Japanese population: The Hisayama Study, Nakamura 1899–1907
Elevated serum pentosidine is independently associated with the high prevalence of sarcopenia in Chinese middle‐aged and elderly men with type 2 diabetes mellitus, Zhang 2054–2061

Sarcopenia and obesity

Association of mean corpuscular volume with sarcopenia and visceral obesity in individuals without anemia, Tanaka 1287–1292

Saturated fatty acids

Lower intake of saturated fatty acids is associated with persistently higher arterial stiffness in patients with type 2 diabetes, Mita 226–233

ScRNA‐seq

Islet β‐cells physiological difference study of old and young mice based on single‐cell transcriptomics, Zheng 1775–1783

Sedentary behavior

Effects of sedentary behavior and daily walking steps on body mass index and body composition: Prospective observational study using outpatient clinical data of Japanese patients with type 2 diabetes, Nakanishi 1732–1738

Serum progesterone

Serum progesterone and retinopathy in male patients with type 2 diabetes: A cross‐sectional study, Sun 1228–1235

Serum prostate‐specific antigen

Association between serum prostate‐specific antigen concentrations and the risk of developing type 2 diabetes mellitus in Chinese men: A cohort study, Huang 1560–1568

Sex differences

Effect of hypertriglyceridemia in dyslipidemia‐induced impaired glucose tolerance and sex differences in dietary features associated with hypertriglyceridemia among the Japanese population: The Gifu Diabetes Study, Nonoyama 771–780
Sex‐specific differences in blood lipids and lipid ratios in type 2 diabetic foot patients, Yin, 2203–2211

Short stature

Metabolic syndrome coexists with adult Léri–Weill dyschondrosteosis: A case report, Wang 446–449

SHORT syndrome

Novel *PIK3R1* mutation of SHORT syndrome: A case report with a 6‐month follow up, Yin 1919–1922

SIRT7

SIRT7 regulates lipogenesis in adipocytes through deacetylation of PPARγ2, Akter 1765–1774

Skeletal muscle

Role of skeletal muscle lipids in the pathogenesis of insulin resistance of obesity and type 2 diabetes, Gilbert 1934–1941

SLC5A2

Clinical and genetic determinants of urinary glucose excretion in patients with diabetes mellitus, Monobe 728–737

Small fiber neuropathy

Corneal confocal microscopy: A useful tool for diagnosis of small fiber neuropathy in type 2 diabetes, Jin 2183–2189

Social determinants of health

Non‐financial social determinants of diabetes among public assistance recipients in Japan: A cohort study, Nishioka 1104–1111

Social media

Efficacy of lifestyle intervention program for Arab women with prediabetes using social media as an alternative platform of delivery, Al‐Hamdan 1872–1880

Sodium–glucose cotransporter 2

Sodium–glucose cotransporter 2 inhibitors compared with other glucose‐lowering drugs in Japan: Subanalyses of the CVD‐REAL 2 Study, Kohsaka 67–73
Blood glucose levels and bodyweight change after dapagliflozin administration, Kim 1594–1602

Sodium–glucose cotransporter inhibitors

Sodium–glucose cotransporter inhibitors as add‐on therapy in addition to insulin for type 1 diabetes mellitus: A meta‐analysis of randomized controlled trials, Zou 546–556

Sodium–glucose cotransporter 2 inhibitor

Sodium–glucose cotransporter 2 inhibitors represent a paradigm shift in the prevention of heart failure in type 2 diabetes patients Kashiwagi, 6–20
Effects of ipragliflozin versus metformin in combination with sitagliptin on bone and muscle in Japanese patients with type 2 diabetes mellitus: Subanalysis of a prospective, randomized, controlled study (PRIME‐V study), Koshizaka 200–206
Early detection of euglycemic ketoacidosis during thoracic surgery associated with empagliflozin in a patient with type 2 diabetes: A case report, Kitahara 664–667
Favorable effect of sodium–glucose cotransporter 2 inhibitor, dapagliflozin, on non‐alcoholic fatty liver disease compared with pioglitazone, Cho 1272–1277
Impact of endogenous insulin secretion on the improvement of glucose variability in Japanese patients with type 2 diabetes treated with canagliflozin plus teneligliptin, Miya 1395–1399
Improved time in range and postprandial hyperglycemia with canagliflozin in combination with teneligliptin: Secondary analyses of the CALMER study, Cho 1417–1424
Sodium–glucose cotransporter 2 inhibitors in type 2 diabetes patients with renal function impairment slow the annual renal function decline, in a real clinical practice, Hirai 1577–1585
On‐label use of sodium–glucose cotransporter 2 inhibitors might increase the risk of diabetic ketoacidosis in patients with type 1 diabetes, Horii 1586–1593

Sodium–glucose cotransporter 2 inhibitors

Blood pressure after treatment with sodium–glucose cotransporter 2 inhibitors influences renal composite outcome: Analysis using propensity score‐matched models, Kobayashi, 74‐81
Sodium–glucose cotransporter 2 inhibitors reduce day‐to‐day glucose variability in patients with type 1 diabetes, Chiba 176–183
Do sodium–glucose cotransporter 2 inhibitors lead to fracture risk? A pharmacovigilance real‐world study, Zhao 1400–1407
Sodium–glucose cotransporter 2 inhibitor‐induced reduction in the mean arterial pressure improved renal composite outcomes in type 2 diabetes mellitus patients with chronic kidney disease: A propensity score‐matched model analysis in Japan, Kobayashi 1408–1416
Incidence of adverse cardiovascular events in type 2 diabetes mellitus patients after initiation of glucose‐lowering agents: A population‐based community study from the Shizuoka Kokuho database, Kohsaka 1452–1461

Sodium–glucose cotransporters

Diet intake control is indispensable for the gluconeogenic response to sodium–glucose cotransporter 2 inhibition in male mice, Hashiuchi 35–47

Spontaneous discitis

Pyogenic psoas abscess on the dorsal side, and bacterial meningitis and spinal epidural abscess on the ventral side, both of which were induced by spontaneous discitis in a patient with diabetes mellitus: A case report, Horiya 1301–1305

Stable coronary artery disease

Impact of daily glucose fluctuations on cardiovascular outcomes after percutaneous coronary intervention for patients with stable coronary artery disease undergoing lipid‐lowering therapy, Yamamoto 1015–1024

Statins

Forty‐eight weeks of statin therapy for type 2 diabetes mellitus patients with lower extremity atherosclerotic disease: Comparison of the effects of pitavastatin and atorvastatin on lower femoral total plaque areas, Zhou 1278–1286

Streptozotocin‐induced diabetes

Calcium signaling in endocardial and epicardial ventricular myocytes from streptozotocin‐induced diabetic rats, Al Kury 493–500

Subclinical myocardial remodeling

Association of the Non‐Alcoholic Fatty Liver Disease Fibrosis Score with subclinical myocardial remodeling in patients with type 2 diabetes: A cross‐sectional study in China, Fan 1035–1041

Subcutaneous insulin resistance syndrome

Case of type 2 diabetes mellitus with edema resulting in subcutaneous insulin resistance syndrome Ying Hu, Qiongwen Zhu, Xiuli Yang, Jieping Yan, and Jiana Shi 2267–2270

Surgery

Preoperative fundus examination in patients with diabetes scheduled for surgery, Watanabe 1508–1511

Sympathovagal effects

Metabolic and sympathovagal effects of bolus insulin glulisine versus basal insulin glargine therapy in people with type 2 diabetes: A randomized controlled study, Takeshita 1193–1201

Systolic blood pressure

Asymptomatic postprandial hypotension in patients with diabetes: The KAMOGAWA‐HBP study, Kitae 837–844

Tartrate‐resistant acid phosphatase‐5b

Lower bone mineral density and higher bone resorption marker levels in premenopausal women with type 1 diabetes in Japan, Yoshioka 1689–1696

Tear

Association between tear and blood glucose concentrations: Random intercept model adjusted with confounders in tear samples negative for occult blood, Aihara 266–276

Tetracycline‐regulated expression

Using recombinase‐mediated cassette exchange to engineer MIN6 insulin‐secreting cells based on a newly identified safe harbor locus, Furukawa 2129–2140

Three‐vessel disease

Prediabetes and long‐term outcomes in patients with three‐vessel coronary artery disease: A large single‐center cohort study, Yuan 409–416

Time in range

Association of time in range, as assessed by continuous glucose monitoring, with painful diabetic polyneuropathy, Yang 828–836
Association of time in range with hemoglobin A1c, glycated albumin and 1,5‐anhydro‐d‐glucitol, Ohigashi 940–949
Improved time in range and postprandial hyperglycemia with canagliflozin in combination with teneligliptin: Secondary analyses of the CALMER study, Cho 1417–1424

Tofogliflozin

Safety and effectiveness of tofogliflozin in Japanese patients with type 2 diabetes mellitus treated in real‐world clinical practice: Results of a 36‐month post‐marketing surveillance study (J‐STEP/LT), Utsunomiya 184–199

Transient receptor potential cation channel subfamily M member 5

Novel association between a transient receptor potential cation channel subfamily M member 5 expression quantitative trait locus rs35197079 and decreased susceptibility of gestational diabetes mellitus in a Chinese population, Li 2062–2070

Trichloroacetic acid

Combination therapy of trichloroacetic acid, human autologous fibroblast injection and fibroblast seeded microfibrous collagen scaffold as a novel treatment for osteomyelitis diabetic foot ulcer, Nilforoushzadeh 1112–1117

Triglyceride–glucose index

Association between the triglyceride–glucose index and diabetic nephropathy in patients with type 2 diabetes: A cross‐sectional study, Liu 557–565

Triglyceride to high‐density lipoprotein cholesterol ratio

Triglyceride to high‐density lipoprotein cholesterol ratio variability and incident diabetes: A 7‐year prospective study in a Chinese population, Cai 1864–1871

Tuberculosis

Metformin as a potential protective therapy against tuberculosis in patients with diabetes mellitus: A retrospective cohort study in a single teaching hospital, Fu 1603–1609

Tumor necrosis factor receptor

Fractional excretion of tumor necrosis factor receptor 1 and 2 in patients with type 2 diabetes and normal renal function, Gohda 382–389

Type 1 and type 2 diabetes

High prevalence and clinical impact of dynapenia and sarcopenia in Japanese patients with type 1 and type 2 diabetes: Findings from the Impact of Diabetes Mellitus on Dynapenia study, Mori 1050–1059

Type 1 collagen

Glyoxal‐induced formation of advanced glycation end‐products in type 1 collagen decreases both its strength and flexibility *in vitro*, Kitamura 1555–1559

Type 1 diabetes

Microencapsulation of cells and molecular therapy of type 1 diabetes mellitus: The actual state and future perspectives between promise and progress, Basta 301–309
Is it possible to predict the onset of nocturnal asymptomatic hypoglycemia in patients with type 1 diabetes receiving insulin degludec? Potential role of previous day and next morning glucose values, Takahashi 365–373
Safely finishing a half marathon by an adult with type 1 diabetes using a commercially available hybrid closed‐loop system, van den Boom 450–453
Different interaction of onset age and duration of type 1 diabetes on the dynamics of autoantibodies to insulinoma‐associated antigen‐2 and zinc transporter 8, Kawasaki 510–515
Young people with type 1 diabetes on insulin pump therapy could fast safely during COVID–19 pandemic Ramadan: A telemonitoring experience in Bangladesh, Zabeen 1060–1063
Impact of glucagon response on early postprandial glucose excursions irrespective of residual β‐cell function in type 1 diabetes: A cross‐sectional study using a mixed meal tolerance test, Ito 1367–1376
Predictors of the maximal oxygen consumption in adult patients with type 1 diabetes treated with personal insulin pumps, Matejko 1377–1385
Epidemiology, genetic landscape and classifi cation of childhood diabetes mellitus in the State of Qatarm Haris 2141–2148

Type 1 diabetes mellitus

Type 1 diabetes management and outcomes: A multicenter study in Thailand, Dejkhamron 516–526
Diagnostic value of combined islet antigen‐reactive T cells and autoantibodies assays for type 1 diabetes mellitus, Tang 963–969
On‐label use of sodium–glucose cotransporter 2 inhibitors might increase the risk of diabetic ketoacidosis in patients with type 1 diabetes, Horii 1586–1593
Lower bone mineral density and higher bone resorption marker levels in premenopausal women with type 1 diabetes in Japan, Yoshioka 1689–1696
Glycemic control in children and teenagers with type 1 diabetes around lockdown for COVID‐19: A continuous glucose monitoring‐based observational study, Wu 1708–1717

Type 2 diabetes

Sodium–glucose cotransporter 2 inhibitors compared with other glucose‐lowering drugs in Japan: Subanalyses of the CVD‐REAL 2 Study, Kohsaka 67–73
Effect of the FreeStyle Libre™ flash glucose monitoring system on glycemic control in individuals with type 2 diabetes treated with basal–bolus insulin therapy: An open label, prospective, multicenter trial in Japan, Ogawa 82–90
Sex differences in sarcopenia and frailty among community‐dwelling Korean older adults with diabetes: The Korean Frailty and Aging Cohort Study, Kang 155–164
Relationships between time in range, glycemic variability including hypoglycemia and types of diabetes therapy in Japanese patients with type 2 diabetes mellitus: Hyogo Diabetes Hypoglycemia Cognition Complications study, Kuroda 244–253
Health‐related quality of life and its predictors among the type 2 diabetes population of Bangladesh: A nation‐wide cross‐sectional study, Barua 277–285
Significance of body mass index for diagnosing sarcopenia is equivalent to slow gait speed in Japanese individuals with type 2 diabetes: Cross‐sectional study using outpatient clinical data, Nakanishi 417–424
Dietary fiber intake and risk of type 2 diabetes in a general Japanese population: The Hisayama Study, Kimura 527–536
Associations between urinary 6‐sulfatoxymelatonin excretion and diabetic vascular complications or arteriosclerosis in patients with type 2 diabetes, Tanaka 601–609
Development of a clinical risk score for incident diabetes: A 10‐year prospective cohort study, Oh 610–618
Biliary diversion increases resting energy expenditure leading to decreased blood glucose level in mice with type 2 diabetes, Yin 931–939
Glucose‐lowering action through targeting islet dysfunction in type 2 diabetes: Focus on dipeptidyl peptidase‐4 inhibition, Ahrén 1128–1135
Cardiovascular events and mortality in people with and without type 2 diabetes: An observational study in a contemporary multi‐ethnic population, Coles 1175–1182
Normal parathyroid hormone and non‐proliferative diabetic retinopathy in patients with type 2 diabetes, Sun 1220–1227
Decreased plasma n6 : n3 polyunsaturated fatty acids ratio interacting with high C‐peptide promotes non‐alcoholic fatty liver disease in type 2 diabetes patients, Luo 1263–1271
Favorable effect of sodium–glucose cotransporter 2 inhibitor, dapagliflozin, on non‐alcoholic fatty liver disease compared with pioglitazone, Cho 1272–1277
Beneficial effects of switching to denosumab from bisphosphonates or selective estrogen receptor modulators in postmenopausal women with type 2 diabetes and osteopenia/osteoporosis, Miyoshi 1293–1300
Identification of two novel subgroups in patients with diabetes mellitus and their association with clinical outcomes: A two‐step cluster analysis, Xiong 1346–1358
Association of serum CTRP9 levels with cardiac autonomic neuropathy in patients with type 2 diabetes mellitus, Yang 1442–1451
Association of plant‐based diet and type 2 diabetes mellitus in Chinese rural adults: The Henan Rural Cohort Study, Yang 1569–1576
Insulin resistance is independently associated with cardiovascular autonomic neuropathy in type 2 diabetes, Liu 1651–1662
Association of hip fractures with cardiometabolic‐renal risk factors in Southern Chinese patients with type 2 diabetes – the Hong Kong Diabetes Register, Cheung 1739–1748
Valuing health states of people with type 2 diabetes: Analyses of the nationwide representative linked databases, Kuo 1749–1758
Simplification of complex insulin regimens using canagliflozin or liraglutide in patients with well‐controlled type 2 diabetes: A 24‐week randomized controlled trial, Ando 1816–1826
Combination of disease duration‐to‐age at diagnosis and hemoglobin A1c‐to‐serum C‐peptide reactivity ratios predicts patient response to glucose‐lowering medication in type 2 diabetes: A retrospective cohort study across Japan (JDDM59), Kanatsuka 1967–1977
iGlarLixi reduces residual hyperglycemia in Japanese patients with type 2 diabetes uncontrolled on basal insulin: A post‐hoc analysis of the LixiLan JP‐L trial, Yabe 1992–2001
Islet microangiopathy and augmented β‐cell loss in Japanese non‐obese type 2 diabetes patients who died of acute myocardial infarction, Takahashi 2149–2161
Corneal confocal microscopy: A useful tool for diagnosis of small fiber neuropathy in type 2 diabetes, Jin 2183–2189
Association of visit‐to‐visit glycemic variability with risk of cardiovascular diseases in high‐risk Japanese patients with type 2 diabetes: A subanalysis of the EMPATHY trial, Sato 2190–2196
Association between Grit Scales and adherence to regular hospital visits among Japanese patients with type 2 diabetes: Prospective observational study, Nakanishi 2259–2262

Type 2 diabetes mellitus

Sodium–glucose cotransporter 2 inhibitors represent a paradigm shift in the prevention of heart failure in type 2 diabetes patients Kashiwagi, 6–20
Antiplatelet strategy in primary and secondary prevention of cardiovascular disease in patients with type 2 diabetes mellitus: A perspective from the guideline appraisal, Liu 99–108
Correlation analysis of microribonucleic acid‐155 and microribonucleic acid‐29 with type 2 diabetes mellitus, and the prediction and verification of target genes, Zhu 165–175
Safety and effectiveness of tofogliflozin in Japanese patients with type 2 diabetes mellitus treated in real‐world clinical practice: Results of a 36‐month post‐marketing surveillance study (J‐STEP/LT), Utsunomiya 184–199
Impact of primary aldosteronism on renal function in patients with type 2 diabetes, Katsuragawa 217–225
Micro‐ribonucleic acid‐23a‐3p prevents the onset of type 2 diabetes mellitus by suppressing the activation of nucleotide‐binding oligomerization‐like receptor family pyrin domain containing 3 inflammatory bodies‐caused pyroptosis through negatively regulating NIMA‐related kinase 7, Chang 334–345
Changing epidemiology of chronic kidney disease as a result of type 2 diabetes mellitus from 1990 to 2017: Estimates from Global Burden of Disease 2017, Li 346–356
Phase III, randomized, double‐blind, placebo‐controlled study to evaluate the efficacy and safety of teneligliptin monotherapy in Chinese patients with type 2 diabetes mellitus inadequately controlled with diet and exercise, Ji 537–545
Diagnostic utility of corneal confocal microscopy in type 2 diabetic peripheral neuropathy, Wang 574–582
Potential biomarker in serum for predicting susceptibility to type 2 diabetes mellitus: Free fatty acid 22:6, Ma 950–962
Association of the Non‐Alcoholic Fatty Liver Disease Fibrosis Score with subclinical myocardial remodeling in patients with type 2 diabetes: A cross‐sectional study in China, Fan 1035–1041
Association of non‐alcoholic fatty liver disease with diabetic retinopathy in type 2 diabetic patients: A meta‐analysis of observational studies, Song 1471–1479
Plasma xanthine oxidoreductase activity in Japanese patients with type 2 diabetes across hospitalized treatment, Kawachi 1512–1520
Association between serum prostate‐specific antigen concentrations and the risk of developing type 2 diabetes mellitus in Chinese men: A cohort study, Huang 1560–1568
Efficacy and safety of the fixed‐ratio combination of insulin degludec and liraglutide by baseline glycated hemoglobin, body mass index and age in Japanese individuals with type 2 diabetes: A subgroup analysis of two phase III trials, Komatsu 1610–1618
Quantitative vibration perception threshold in assessing diabetic polyneuropathy: Should the cut‐off value be adjusted for Chinese individuals with type 2 diabetes?, Liu 1663–1670
Prospective study: Aldehyde dehydrogenase 2 gene is associated with cardio‐cerebrovascular complications in type 2 diabetes patients, He 1845–1854
Risk of early mortality and cardiovascular disease according to the presence of recently diagnosed diabetes and requirement for insulin treatment: A nationwide study, Lee 1855–1863
Lower insulin secretion is associated with hippocampal and parahippocampal gyrus atrophy in elderly patients with type 2 diabetes mellitus, Adachi 1908–1913
Case of type 2 diabetes mellitus with edema resulting in subcutaneous insulin resistance syndrome Ying Hu, Qiongwen Zhu, Xiuli Yang, Jieping Yan, and Jiana Shi 2267–2270

Type 2 diabetes mortality

Thresholds for postprandial hyperglycemia and hypertriglyceridemia associated with increased mortality risk in type 2 diabetes patients: A real‐world longitudinal study, Takao 886–893

Type 2 diabetes prevention

Sex differences in the association between fatty liver and type 2 diabetes incidence in non‐obese Japanese: A retrospective cohort study, Narisada 1480–1489

Type of family doctor

Preoperative fundus examination in patients with diabetes scheduled for surgery, Watanabe 1508–1511

Underweight

All‐cause and cardiovascular disease mortality in underweight patients with diabetic nephropathy: BioBank Japan cohort, Yokomichi 1425–1429

Uptake

Screening for diabetic retinopathy with different levels of financial incentive in a randomized controlled trial, Lian 1632–1641

Urothelial carcinoma‐associated 1

Long non‐coding ribonucleic acid urothelial carcinoma‐associated 1 promotes high glucose‐induced human retinal endothelial cells angiogenesis through regulating micro‐ribonucleic acid‐624‐3p/vascular endothelial growth factor C, Yan 1948–1957

Variability

Triglyceride to high‐density lipoprotein cholesterol ratio variability and incident diabetes: A 7‐year prospective study in a Chinese population, Cai 1864–1871

Vaspin

Serum vaspin levels are positively associated with diabetic retinopathy in patients with type 2 diabetes mellitus, Yang 566–573

Vibration perception threshold

Quantitative vibration perception threshold in assessing diabetic polyneuropathy: Should the cut‐off value be adjusted for Chinese individuals with type 2 diabetes?, Liu 1663–1670

Visit‐to‐visit

Mean and visit‐to‐visit variability of glycated hemoglobin, and the risk of non‐alcoholic fatty liver disease, Yoo 1252–1262

Vitamin D

Vitamin D affects the neutrophil‐to‐lymphocyte ratio in patients with type 2 diabetes mellitus, Wang 254–265

Walking steps

Effects of sedentary behavior and daily walking steps on body mass index and body composition: Prospective observational study using outpatient clinical data of Japanese patients with type 2 diabetes, Nakanishi 1732–1738

Weight

Blood glucose levels and bodyweight change after dapagliflozin administration, Kim 1594–1602

Weight reduction therapy

Association of protein tyrosine phosphatase 1B gene polymorphism with the effects of weight reduction therapy on bodyweight and glycolipid profiles in obese patients, Yamakage 1462–1470

Xanthine oxidoreductase

Plasma xanthine oxidoreductase activity in Japanese patients with type 2 diabetes across hospitalized treatment, Kawachi 1512–1520

Zebrafish

Effect of adenosine monophosphate‐activated protein kinase–p53–Krüppel‐like factor 2a pathway in hyperglycemia‐induced cardiac remodeling in adult zebrafish, Wang 320–333
Investigating the role of *dachshund b* in the development of the pancreatic islet in zebrafish, Yang 710–727

